# Supplementary material for: Sterile inflammation via TRPM8 RNA-dependent TLR3-NF-kB/IRF3 activation promotes antitumor immunity in prostate cancer
Source: EMBO J. 2024 Feb 5;43(5):6. doi: 10.1038/s44318-024-00040-5 (PMC10907604; doi:10.1038/s44318-024-00040-5)
Supplement: Supplementary file 1 — Table EV1 [file 44318_2024_40_MOESM1_ESM.docx]

**Table EV1: Description of primers used in this study**

| ***Human Genes*** | ***Forward*** | ***Reverse*** |
| --- | --- | --- |
| *BAG6* | TTGGTGAAGACCTTGGACTC | TTCAGATGGGATGCTGACAG |
| *COLGALT1* | ACTCCACGGAATGGTACAAAC | CTACGGACCACAACATGGATAAC |
| *COLGALT2* | CTCCAATGGAGCCTTCCCAGTT | TCTCCTGTTCGTAGAGTGTGCG |
| *CXCL1* | CGCCCAAACCGAAGTCAT | TAACTATGGGGGATGCAGGA |
| *CXCL10* | TTCAAGGAGTACCTCTCTCTAG | CTGGATTCAGACATCTCTTCTC |
| *GAPDH* | TGAAGGTCGGAGTCAACGGATTTGG | CATGTGGGCCATGAGGTCCACCAC |
| *IF-β* | AGGACAGGATGAACTTTGAC | TGATAGACATTAGCCAGGAG |
| *IF-γ* | TCAGCTCTGCATCGTTTTGG | GTTCCATTATCCGCTACATCTGAA |
| *IL1b* | GGACAGGATATGGAGCAAC | AGCTGTAGAGTGGGCTTATC |
| *IL6* | TACATCCTCGACGGCATCTC | CCATCTTTGGAAGGTTCAGG |
| *KLK2* | CCACACCCGCTCTACAATATG | GAAGCACACCATTACAGACAAG |
| *KLK3* | CCTCCTGAAGAATCGATTCCT | GAGGTCCACACACTGAAGTT |
| *NCR3LG1* | ACCCTGGGACTGTCTACCAG | TGAAATAGGCCACCAATGAA |
| *NKX3.1* | ACGTCCTTCCTCATCCAGGACA | AGGGCGCCTGAAGTGTTTTC |
| *P3H3* | GACTGCCTGACCCAGTGC | CTGCCAGATCCAGCTTCTTC |
| *P3H4* | CATGAGCAGGTGGACTTCAAGG | ACTTGTCCACGAAGTAGCCACC |
| *TMPRSS2* | TCCTTCAGGTGTACTCATCTC | GCTGTCATCCACTATTCCTTG |
| *TRPM8 Ex4-5* | CGGAGAATGTGTGCAAGTGT | ATGTAGATGAGCCGGCTGAA |
| *TRPM8 Ex8-9* | CTGTCATGGACATCCCACTG | GGGATCTTGCCACCATAGTT |
| *TRPM8 Ex12-15* | GATTTTCACCAATGACCGCCG | CCCCAGCAGCATTGATGTCG |
| *TRPM8 Ex15-18* | GGTCTATTCCTGTGAAGCTTG | CCAGCGTGTCCATCACATTC |
| *TRPM8 Ex21-23* | CCACGTATGACTTTGCCCAC | TTCCTTCATGACACCCTCCC |
| *Y3* | GGCTGGTCCGAGTGCAGTG | GAAGCAGTGGGAGTGGAGAA |
