# Supplementary material for: Sterile inflammation via TRPM8 RNA-dependent TLR3-NF-kB/IRF3 activation promotes antitumor immunity in prostate cancer
Source: EMBO J. 2024 Feb 5;43(5):6. doi: 10.1038/s44318-024-00040-5 (PMC10907604; doi:10.1038/s44318-024-00040-5)
Supplement: Supplementary file 2 — Table EV2 [file 44318_2024_40_MOESM2_ESM.docx]

**Table EV2: List of antibodies used in this study**

| ***Primary Antibody*** | ***Supplier*** | ***Catalog #*** | ***Type*** | ***WB***  ***Dilution*** | ***IP*** | ***IF/IHC Dilution*** |
| --- | --- | --- | --- | --- | --- | --- |
| AR | Santa Cruz | sc-816 | Rabbit pAb | 1:200 - 1:1000 |  |  |
| AR | Santa Cruz | sc-7305 | Mouse mAb | 1:500 |  |  |
| B220 | Serotec | MCA1258G | Rat mAb |  |  | FFPE 1:750 |
| Cleaved Caspase3 | CST | 9661 | Rabbit pAb | 1:500 |  |  |
| COL1A1 | CST | 91144 | Rabbit mAb | 1:1000 |  |  |
| IBA1 | Fujifilm Wako | 019-19741 | Rabbit pAb |  |  | FFPE 1:1000 |
| IRF3 | CST | 11904 | Rabbit mAb | 1:1000 |  |  |
| Fibrillarin | Abcam | ab4566 | Mouse mAb | 1:1000 |  |  |
| GAPDH | Life Tech | MA515738 | Mouse mAb | 1:5000 |  |  |
| MHCII | Novus Bio | NBP1-43312 | Rat mAb |  |  | FFPE 1:200 |
| NF-kB p65 | CST | 8242 | Rabbit mAb | 1:1000 |  | IF 1:200 |
| NKp46/NCR1 | R&D Systems | AF2225 | Goat pAb |  |  | FFPE 1:200 |
| NKp46/NCR1 | Biolegend | 137602 | Rat mAb |  |  | FFPE 1:200 |
| NKX3.1 | Millipore | AB5983 | Rabbit pAb | 1:1000 |  |  |
| PARP | CST | 9542 | Rabbit pAb | 1:1000 |  |  |
| Phospho-P50 | Santa Cruz | Sc-271908 | Mouse mAb | 1:1000 |  |  |
| cRel | CST | 4727 | Rabbit pAb | 1:1000 |  |  |
| PSA | Invitrogen | PA1-38514 | Rabbit pAb |  |  | FFPE 1:200 |
| PSA | Dako | M0750 | Mouse mAb | 1:500 |  |  |
| KLK2 | Origene | TA802077 | Mouse mAb | 1:500 |  |  |
| STAT1 | CST | 14994 | Rabbit mAb | 1:1000 |  |  |
| TLR3 | Novus Biol | NB100-56571 | Rabbit pAb |  | 1 μg |  |
| TLR3 | Novus Biol | NBP2-25875 | Mouse mAb | 1:1000 |  |  |
| TRPM8 | Abcam | ab3243 | Rabbit pAb | 1:1000 |  |  |
| TRPM8 | Alomone Labs | ACC-049 | Rabbit pAb | 1:1000 |  |  |
| Vimentin | CST | 5741 | Rabbit mAb | 1:1000 |  | FFPE 1:500 |
| α-SMA | Life Tech | 14-9760-80 | Mouse mAb | 1:200 |  | FFPE 1:2000 |
| β-Actin | Sigma | A2228 | Mouse mAb | 1:4000 |  |  |
| β-Tubulin | Santa Cruz | sc-5274 | Mouse mAb | 1:4000 |  |  |

FFPE: Formalin Fixed Paraffin Embedded sections
