# Supplementary material for: Sterile inflammation via TRPM8 RNA-dependent TLR3-NF-kB/IRF3 activation promotes antitumor immunity in prostate cancer
Source: EMBO J. 2024 Feb 5;43(5):6. doi: 10.1038/s44318-024-00040-5 (PMC10907604; doi:10.1038/s44318-024-00040-5)
Supplement: Supplementary file 3 — Appendix [file 44318_2024_40_MOESM3_ESM.pdf]

Sterile inflammation via TRPM8 RNA-dependent TLR3-NF-kB/IRF3 activation promotes antitumor immunity in prostate cancer

Alessandro Alaimo[1,*], Sacha Genovesi[1], Nicole Annesi[1], Dario De Felice[1], Saurav Subedi[2], Alice Macchia[1], Federico La Manna[2], Yari Ciani[1], Federico Vannuccini[1], Vera Mugoni[1], Michela Notarangelo[1], Michela Libergoli[1], Francesca Broso[1], Riccardo Taulli[3,4], Ugo Ala[5], Aurora Savino[6], Martina Cortese[1], Somayeh Mirzaaghaei[6,7], Valeria Poli[6,7], Ian Marc Bonapace[8], Mauro Giulio Papotti[9], Luca Molinaro[9], Claudio Doglioni[10], Orazio Caffo[11], Adriano Anesi[12], Michael Nagler[13], Giovanni Bertalot[14,15], Francesco Giuseppe Carbone[14], Mattia Barbareschi[14,15], Umberto Basso[16], Erik Dassi[1], Massimo Pizzato[1], Alessandro Romanel[1], Francesca Demichelis[1], Marianna Kruithof-de Julio[2,13], Andrea Lunardi[1,*].

[1]Department of Cellular, Computational and Integrative Biology (CIBIO), University of Trento, Trento, Italy; [2]Department for BioMedical Research, Urology Research Laboratory, University of Bern, Bern, Switzerland; [3]Department of Oncology, University of Torino, Torino, Italy; [4]Center for Experimental Research and Medical Studies (CeRMS), AOU Città della Salute e della Scienza di Torino, Torino, Italy; [5]Department of Veterinary Sciences, University of Torino, Torino, Italy; [6]Department of Molecular Biotechnology and Health Sciences, University of Torino, Torino, Italy; [7]Molecular Biotechnology Center (MBC) "Guido Tarone", University of Torino, Torino, Italy; [8]Department of Biotechnology and Life Sciences, University of Insubria, Busto Arsizio (VA), Italy; [9]Department of Pathology, University of Torino and AOU Città della Salute e della Scienza di Torino, Torino, Italy; [10]Division of Pathology, Pancreas Translational and Clinical Research Center, San Raffaele Scientific Institute IRCCS Vita Salute, San Raffaele University, Milano, Italy; [11]Medical Oncology Department, Santa Chiara Hospital-APSS, Trento, Italy; [12]Operative Unit of Clinical Pathology, Santa Chiara Hospital-APSS, Trento, Italy; [13]Department of Urology, Inselspital, Bern University Hospital, University of Bern, Bern, Switzerland; [14]Operative Unit of Anatomy Pathology, Santa Chiara Hospital-APSS, Trento, Italy; [15]Centre for Medical Sciences-CISMed, University of Trento, Italy; [16]Oncology 1 Unit, Department of Oncology, Istituto Oncologico Veneto IOV IRCCS, Padova, Italy.

*These authors contributed equally to this work.

Corresponding Author: Alessandro Alaimo, Department of Cellular, Computational and Integrative Biology (CIBIO), University of Trento, Via Sommarive 9, 38123 Trento (TN), Italy. Phone:0039-0461-285288; Fax +39 0461 283937. E-mail: alessandro.alaimo@unitn.it; and Andrea Lunardi, Department of Cellular, Computational and Integrative Biology (CIBIO), University of Trento, Via Sommarive 9, 38123 Trento (TN), Italy. Phone:0039-0461-285288; Fax +39 0461 283937. E-mail: andrea.lunardi@unitn.it.

Table of Content:

Appendix Figure S1. *Unaltered features of normal and inflamed prostate cancer cells…2*

Appendix Figure S2. *Quantitation of TRPM8 RNA binding to TLR3………………………..3*

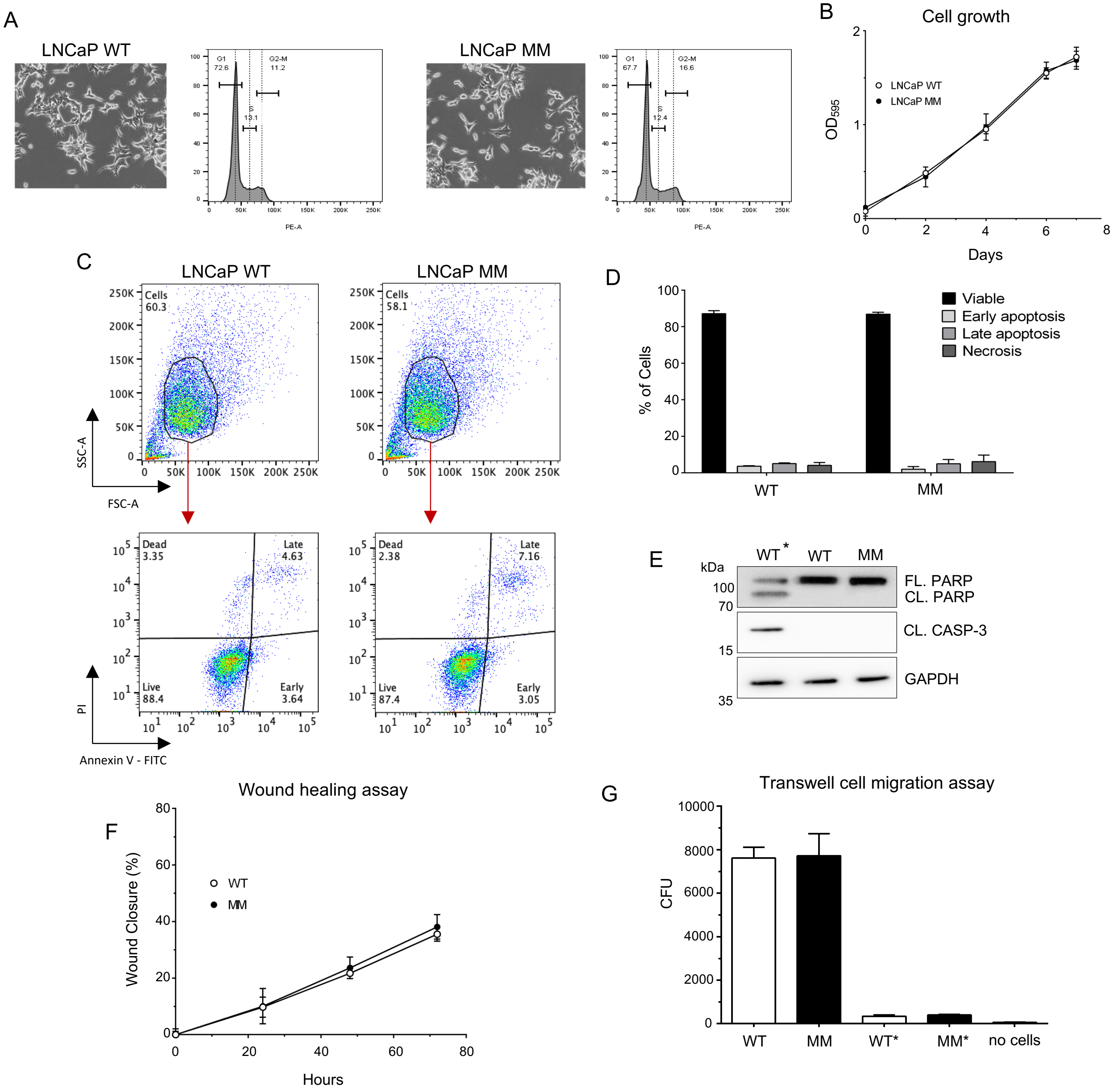

Appendix Figure S1. *Unaltered features of normal and inflammed prostate cancer cells*

A, Morphology (left) and cell cycle analysis by flow cytometry (right) of LNCaP WT and LNCaP MM cell lines (LNCaP WT % of cells in G1 = 72,6; S = 13,1; G2/M = 11,2; LNCaP MM % of cells in G1 = 67,7; S = 12,4; G2/M = 16,6).

B, Cell growth analysis of LNCaP WT and LNCaP MM cell lines.

C and **D,** Cell viability analysis by flow cytometry with Annexin-V/PI staining (C) and relative quantification (D) of LNCaP WT and LNCaP MM cell lines.

E, Cell death study by Western blot analysis of full-length and cleaved PARP and cleaved Caspase 3 LNCaP WT and LNCaP MM cell lines. Staurosporin treatment (1 μM, 6 hours) of WT LNCaP (WT*) was used as positive control of apoptotic cell death. GAPDH was used as loading control.

F and **G**, Wound healing (F) and Transwell migration assays (G, *migrated cells without chemoattractants) of LNCaP WT and LNCaP MM cell lines.

A

RNA	Native RIP			fCLIP		
	LNCaP			LNCaP		
	MM	WT	CAS	MM	WT	CAS
**TRPM8 Ex 4-5**	22.89<br>23.02<br>23.39	24.53<br>24.28<br>24.22	37.76<br>N/A<br>N/A	22.85<br>23.56<br>23.35	24.27<br>24.87<br>24.25	N/A<br>N/A<br>38.29
**TRPM8 Ex 8-9**	38.43<br>N/A<br>N/A	N/A<br>39.42<br>N/A	N/A<br>N/A<br>N/A	39.76<br>N/A<br>N/A	N/A<br>N/A<br>N/A	N/A<br>N/A<br>N/A
**TRPM8 Ex 12-15**	22,71<br>23,11<br>23.68	25.14<br>24.33<br>23.43	36.02<br>37.95<br>39.62	22.41<br>24.76<br>22.74	23.48<br>24.22<br>23.81	N/A<br>39.21<br>38.25
**TRPM8 Ex 15-18**	N/A<br>39.75<br>N/A	38.98<br>N/A<br>N/A	N/A<br>N/A<br>N/A	N/A<br>37.87<br>N/A	N/A<br>N/A<br>N/A	N/A<br>N/A<br>N/A
**TRPM8 Ex 21-23**	25.25<br>25.59<br>N/A	25.49<br>25.81<br>25.31	38.18<br>N/A<br>38.48	24.97<br>25.40<br>25.01	25.31<br>25.69<br>25.47	N/A<br>38.98<br>N/A
**GAPDH**	39.54<br>N/A<br>N/A	N/A<br>N/A<br>37.85	N/A<br>N/A<br>N/A	39.16<br>N/A<br>N/A	N/A<br>38.09<br>N/A	N/A<br>N/A<br>N/A
**Y3**	39.42<br>N/A<br>38.58	N/A<br>N/A<br>N/A	N/A<br>N/A<br>N/A	38.56<br>N/A<br>N/A	N/A<br>N/A<br>N/A	N/A<br>N/A<br>N/A

B

Ex 4-5 MM Native RIP
GCACCCAGATCAACCAAAGTGAGAAATGGAACTACAAGAAACACACCAAGGAATTTCCTACCGA
CGCCTTTGGGGATATTCAGTTTGAGACACTGGGGAAGAAAGGGAAGTATATACGTCTGTCCTGC
GACACGGACGCGGAAATCCTTTACGAGCTGCTGACCCAGCACTGGCACCTGAAAACACCCAAC
CTGGTCATTTCTGTGACCGGGGGCGCCAAG

Ex 4-5 WT Native RIP
GAACTACAAGAAACACACCAAGGAATTTCCTACCGACGCCTTTGGGGATATTCAGTTTGAGACA
CTGGGGAAGAAAGGGAAGTATATACGTCTGTCCTGCGACACGGACGCGGAAATCCTTTACGAG
CTGCTGACCCAGCACTGGCACCTGAAAACACCCAACCTGGTCATTTCTGTGACCGGGGGCGCC
AAGAACTTCGCCCTGAAGCCGCGCATGCGCAAGATCTTCAGCCGGCTCATCT

Ex 12-15 MM Native RIP
AGTCATGTTTACGGCTCTCATAAAGGACAGACCCAAGTTTGTCCGCCTCTTTCTGGAGAATGGC
TTGAACCTACGGAAGTTTCTCACCCATGATGTCCTCACTGAACTCTTCTCCAACCACTTCAGCAC
GCTTGTGTACCGGAATCTGCAGATCGCCAAGAATTCCTATAATGATGCCCTCCTCACGTTTGTCT
GGAAACTGGTTGCGAACTTCCGAAGAGGCTTCCGGAAGGAAGACAGAAATGGCCGGGACGAG
ATGGACATAGAACTCCACGACGTGTCTCCTATTACTCGGCACCCCCTGCAAGCTCTCTTCATCT
GGGCCATTCTTCAGAATAAGAAGGAA

Ex 12-15 WT Native RIP
CTGACCTTCAAGAAGTCATGTTTACGGCTCTCATAAAGGACAGACCCAAGTTTGTCCGCCTCTTT
CTGGAGAATGGCTTGAACCTACGGAAGTTTCTCACCCATGATGTCCTCACTGAACTCTTCTCCA
ACCACTTCAGCACGCTTGTGTACCGGAATCTGCAGATCGCCAAGAATTCCTATAATGATGCCCT
CCTCACGTTTGTCTGGAAACTGGTTGCGAACTTCCGAAGAGGCTTCCGGAAGGAAGACAGAAA
TGGCCGGGACGAGATGGACATAGAACTCCACGACGTGTCTCCTATTACTCGGCACCCCCTGCA
AGCTCTCTTCATCTGGGCCATTCTTCAGAATAAGAAGGAACTCTCCAAAGTCATTTGGGAGCAG
ACCAGGGGCTGCACTCTGGCAGCCCTGGGAGCCAGCAAGCTTCTG

Ex 21-23 MM Native RIP
CAGGTGCCCAGTGACGTGGATGGTACCACGTATGACTTTGCCCACTGCACCTTCACTGGGAAT
GAGTCCAAGCCACTGTGTGTGGAGCTGGATGAGCACAACCTGCCCCGGTTCCCCGAGTGGATC
ACCATCCCCCTGGTGTGCATCTACATGTTATCCACCAACATCCTGCTGGTCAACCTGCTGGTCG
CCATGTTTGGCTACACGGTGGGCACCGTCCAGGAGAACAATGACCAGGTCTGGAAGTTCCAGA
GGTACTTCCTGGTGCAGGAGTACTGCAGCCGCCTCAATATCCCCTTCCCCTTCATCGTCTTCGC
TTACTTCTACATGGTGGTGAAGAAGTGCTTCAAGTGTTGCTGCAAGGAGAAAAACATGGAGTCT
TCTGTCTGCTGTTTCAAAAATGAAGACAATGAGACTCTGGCATGG

Ex 21-23 WT Native RIP
TCGGCCAGGTGCCCAGTGACGTGGATGGTACCACGTATGACTTTGCCCACTGCACCTTCACTG
GGAATGAGTCCAAGCCACTGTGTGTGGAGCTGGATGAGCACAACCTGCCCCGGTTCCCCGAGT
GGATCACCATCCCCCTGGTGTGCATCTACATGTTATCCACCAACATCCTGCTGGTCAACCTGCT
GGTCGCCATGTTTGGCTACACGGTGGGCACCGTCCAGGAGAACAATGACCAGGTCTGGAAGTT
CCAGAGGTACTTCCTGGTGCAGGAGTACTGCAGCCGCCTCAATATCCCCTTCCCCTTCATCGTC
TTCGCTTACTTCTACATGGTGGTGAAGAAGTGCTTCAAGTGTTGCTGCAAGGAGAAAAACATGG
AGTCTTCTGTCTGCTGTTTCAAAAATGAAGACAATGAGACTCTG

Ex 4-5 MM fCLIP
GCACCCAGATCAACCAAAGTGAGAAATGGAACTACAAGAAACACACCAAGGAATTTCCTACCGA
CGCCTTTGGGGATATTCAGTTTGAGACACTGGGGAAGAAAGGGAAGTATATACGTCTGTCCTGC
GACACGGACGCGGAAATCCTTTACGAGCTGCTGACCCAGCACTGGCACCTGAAAACACCCAAC
CTGGTCATTTCTGTGACCGGGGGCGCCAAGAACTTCGCCCTGAAGCCGCGCATGCGCAAGATC
TTCAGCCGG

Ex 4-5 WT fCLIP
CAGATCAACCAAAGTGAGAAATGGAACTACAAGAAACACACCAAGGAATTTCCTACCGACGCCT
TTGGGGATATTCAGTTTGAGACACTGGGGAAGAAAGGGAAGTATATACGTCTGTCCTGCGACAC
GGACGCGGAAATCCTTTACGAGCTGCTGACCCAGCACTGGCACCTGAAAACACCCAACCTGGT
CATTTCTGTGACCGGGGGCGCCAAGAACTTCGCCCTGAAGCCGCG

Ex 12-15 MM fCLIP
CTTCAAGAAGTCATGTTTACGGCTCTCATAAAGGACAGACCCAAGTTTGTCCGCCTCTTTCTGG
AGAATGGCTTGAACCTACGGAAGTTTCTCACCCATGATGTCCTCACTGAACTCTTCTCCAACCA
CTTCAGCACGCTTGTGTACCGGAATCTGCAGATCGCCAAGAATTCCTATAATGATGCCCTCCTC
ACGTTTGTCTGGAAACTGGTTGCGAACTTCCGAAGAGGCTTCCGGAAGGAAGACAGAAATGGC
CGGGACGAGATGGACATAGAACTCCACGACGTGTCTCCTATTACTCGGCACCCCCTGCAAGCT
CTCTTCATCTGGGCCATTCTTCAGAATAAGAAGGAACTCTCCAAAGTCATTTGGGAGCAGACCA
GGGGCTGCACTCTGGCAGCCCTGGGAGCCAGCAAGCTTCTGAAG

Ex 12-15 WT fCLIP
AGTCATGTTTACGGCTCTCATAAAGGACAGACCCAAGTTTGTCCGCCTCTTTCTGGAGAATGGC
TTGAACCTACGGAAGTTTCTCACCCATGATGTCCTCACTGAACTCTTCTCCAACCACTTCAGCAC
GCTTGTGTACCGGAATCTGCAGATCGCCAAGAATTCCTATAATGATGCCCTCCTCACGTTTGTCT
GGAAACTGGTTGCGAACTTCCGAAGAGGCTTCCGGAAGGAAGACAGAAATGGCCGGGACGAG
ATGGACATAGAACTCCACGACGTGTCTCCTATTACTCGGCACCCCCTGCAAGCTCTCTTCATCT
GGGCCATTCTTCAGAATAAGAAGGAACTCTCCAAAGTCATTTGGGAGCAGACCAGGGGCTGCA
CTCTGGCAGCCCTGGGAGCCAGCAAGCTTCTGAAGACTCTGGCCA

Ex 21-23 MM fCLIP
CAGGTGCCCAGTGACGTGGATGGTACCACGTATGACTTTGCCCACTGCACCTTCACTGGGAAT
GAGTCCAAGCCACTGTGTGTGGAGCTGGATGAGCACAACCTGCCCCGGTTCCCCGAGTGGATC
ACCATCCCCCTGGTGTGCATCTACATGTTATCCACCAACATCCTGCTGGTCAACCTGCTGGTCG
CCATGTTTGGCTACACGGTGGGCACCGTCCAGGAGAACAATGACCAGGTCTGGAAGTTCCAGA
GGTACTTCCTGGTGCAGGAGTACTGCAGCCGCCTCAATATCCCCTTCCCCTTCATCGTCTTCGC
TTACTTCTACATGGTGGTGAAGAAGTGCTTCAAGTGTTGCTGCAAGGAGAAAAACATGGAGTCT
TCTGTCTGCTGTTTCAAAAATGAAGACAATGAGACTCTGGCATGG

Ex 21-23 WT fCLIP
CAGTGACGTGGATGGTACCACGTATGACTTTGCCCACTGCACCTTCACTGGGAATGAGTCCAA
GCCACTGTGTGTGGAGCTGGATGAGCACAACCTGCCCCGGTTCCCCGAGTGGATCACCATCCC
CCTGGTGTGCATCTACATGTTATCCACCAACATCCTGCTGGTCAACCTGCTGGTCGCCATGTTT
GGCTACACGGTGGGCACCGTCCAGGAGAACAATGACCAGGTCTGGAAGTTCCAGAGGTACTTC
CTGGTGCAGGAGTACTGCAGCCGCCTCAATATCCCCTTCCCCTTCATCGTCTTCGCTTACTTCT
ACATGGTGGTGAAGAAGTGCTTCAAGTGTTGCTGCAAGGAGAAAAACATGGAGTCTTCTGTCTG
CTGTTTCAAAAATGAAGACAATGAGACTCTGGCATGGGAGGGTGT

Appendix Figure S2. *Quantitation of TRPM8 RNA binding to TLR3*

A, Table showing qPCR threshold cycle (CT) of TRPM8, GAPDH and Y3 transcripts RIP and fCLIP experiments performed with the TLR3 antibody NB100-5657 produced by Novus Biological.

B, Amplicons sequence.
