# Supplementary figures and images for: Sterile inflammation via TRPM8 RNA-dependent TLR3-NF-kB/IRF3 activation promotes antitumor immunity in prostate cancer
Source: EMBO J. 2024 Feb 5;43(5):6. doi: 10.1038/s44318-024-00040-5 (PMC10907604; doi:10.1038/s44318-024-00040-5)

Fig 1C

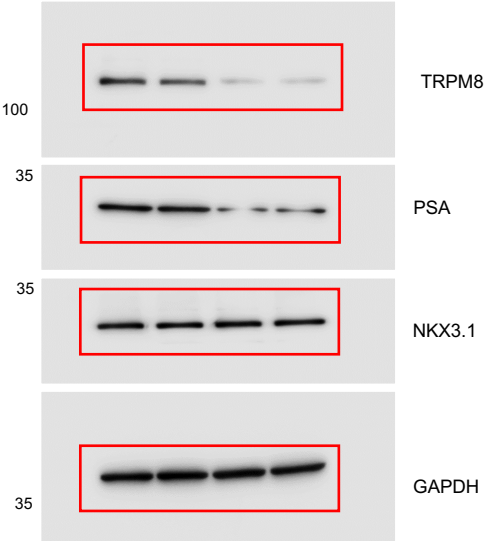

Fig 1F

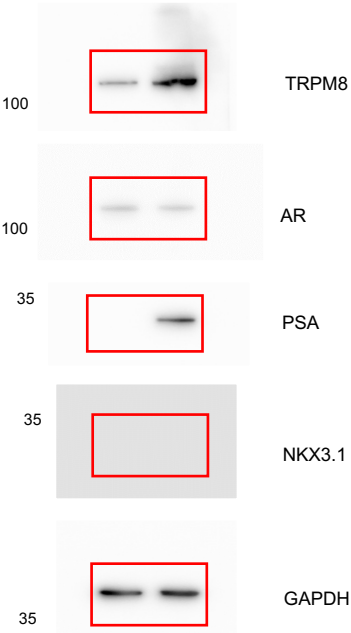

Fig 1H

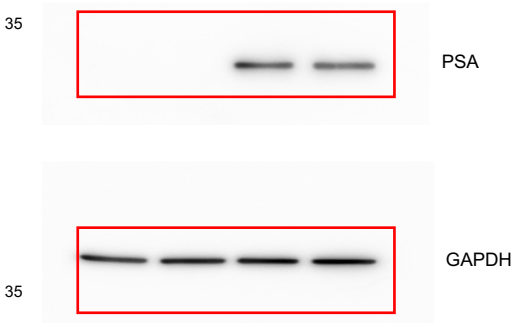

Fig 1I

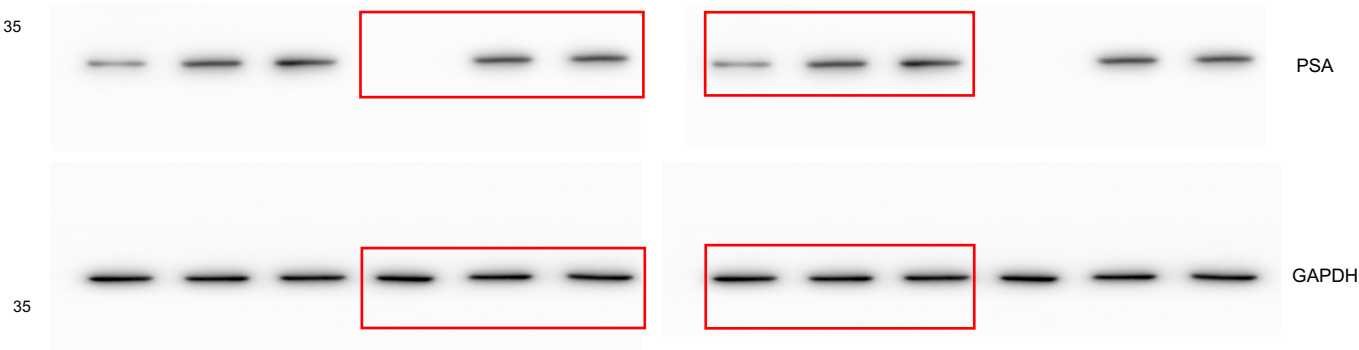

Supplement: Supplementary file 4 — Source Data Fig. 1 [file 44318_2024_40_MOESM4_ESM.zip › Figure 1/Raw Uncropped WB Figure 1.pdf]

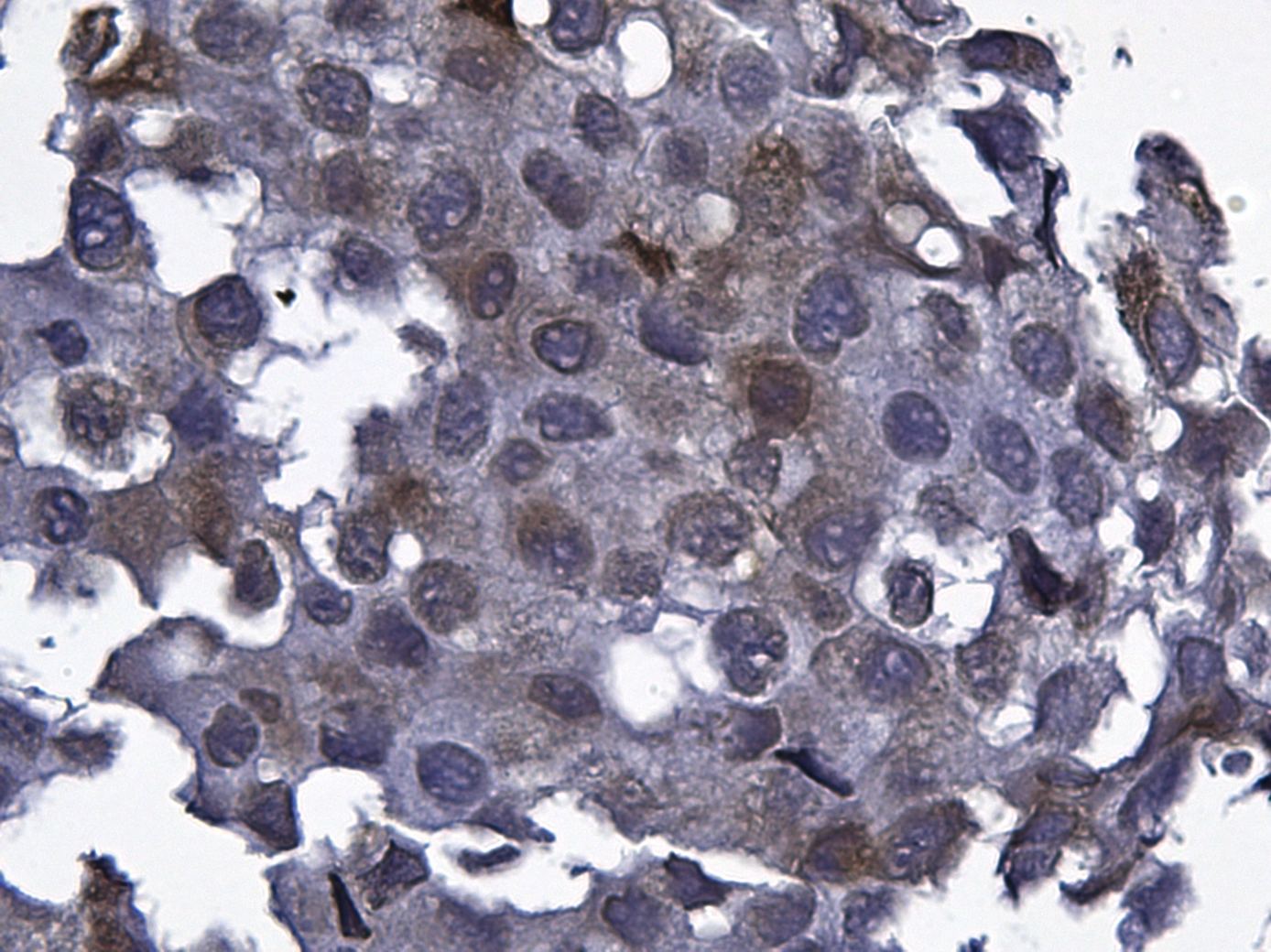

Supplement: Supplementary file 4 — Source Data Fig. 1 [file 44318_2024_40_MOESM4_ESM.zip › Figure 1/1G/1G RWPE-1 M8.TIF]

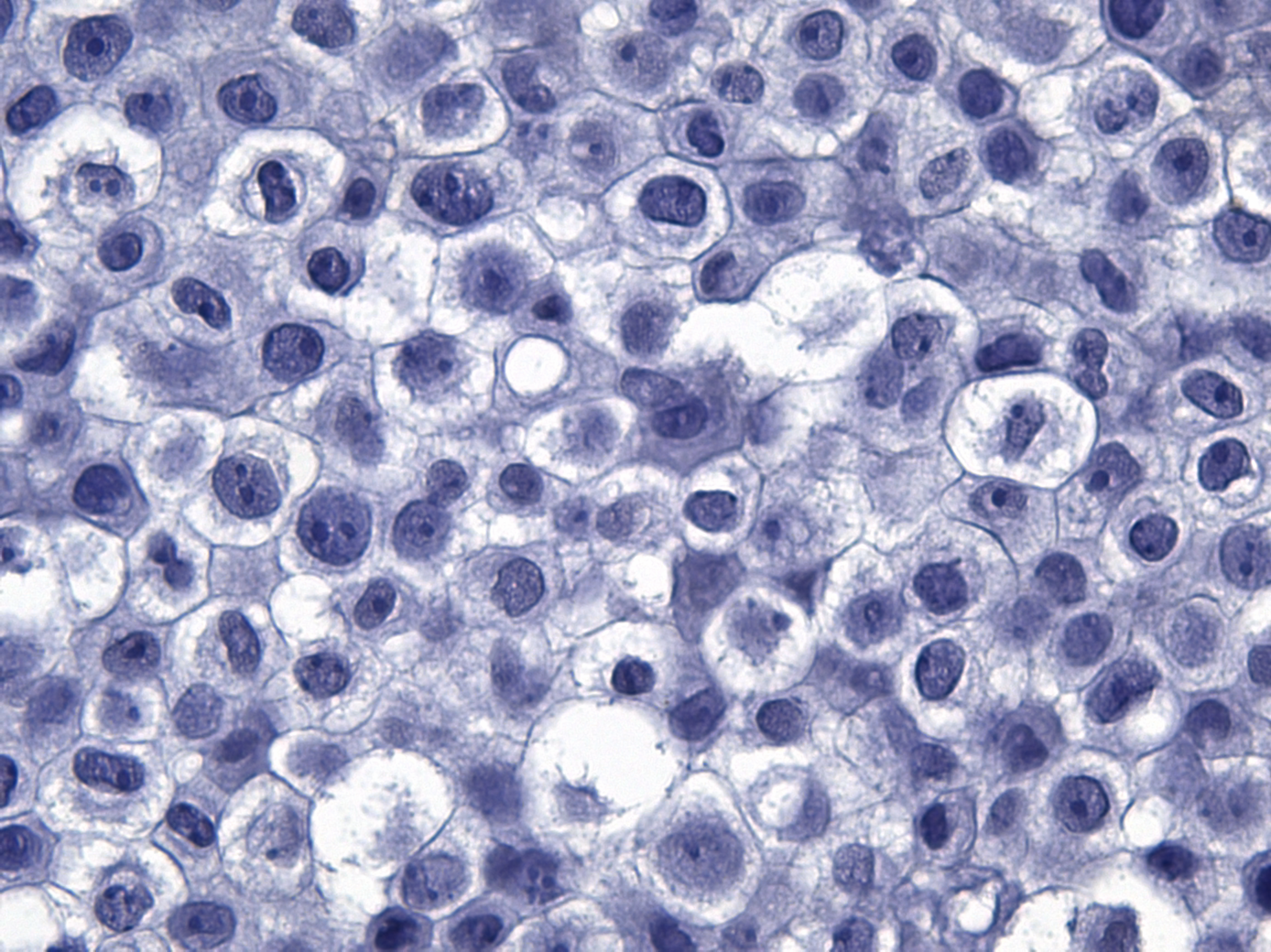

Supplement: Supplementary file 4 — Source Data Fig. 1 [file 44318_2024_40_MOESM4_ESM.zip › Figure 1/1G/1G RWPE WT.TIF]

Fig 2B

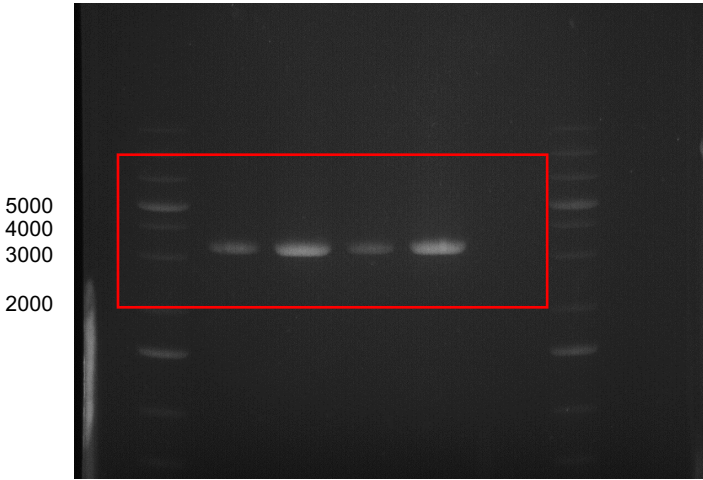

Fig 2E

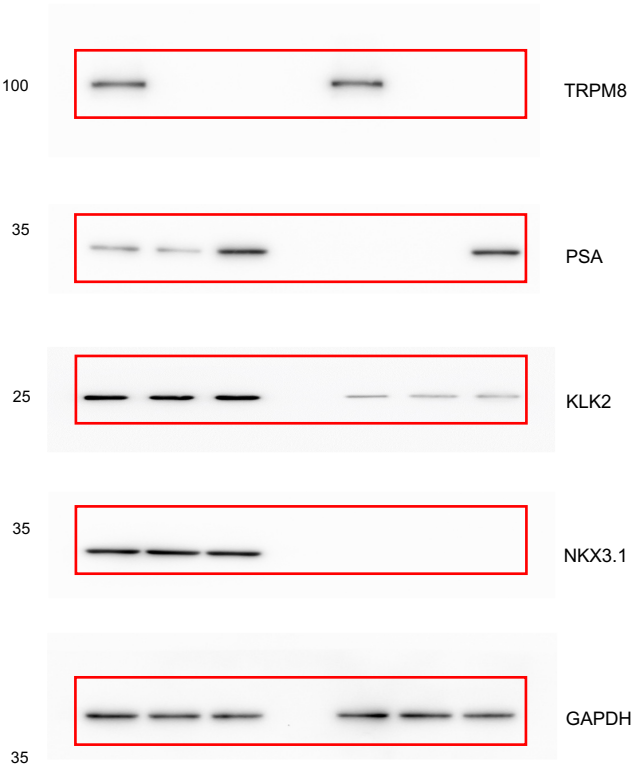

Fig 2

Fig 2G

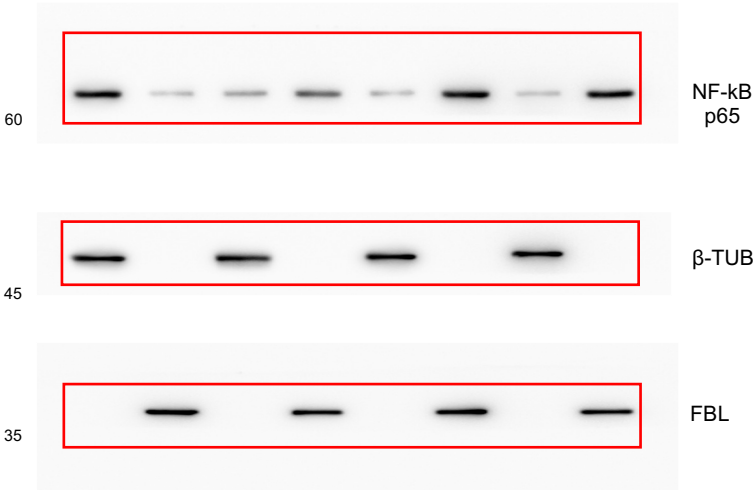

Fig 2H

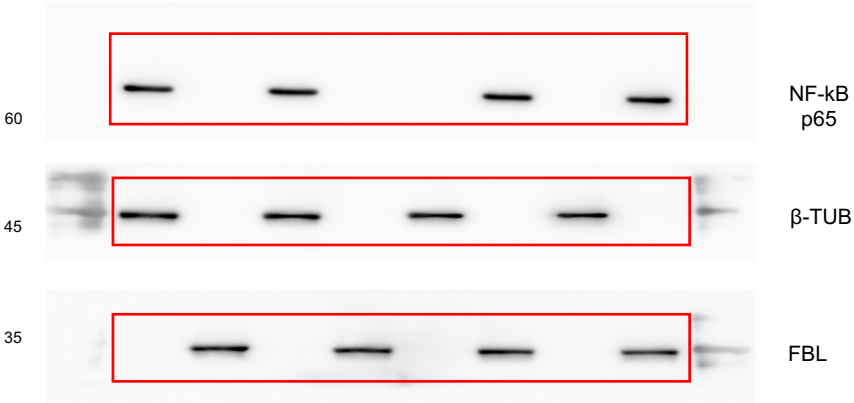

Supplement: Supplementary file 5 — Source Data Fig. 2 [file 44318_2024_40_MOESM5_ESM.zip › Figure 2/Raw Uncropped WB Figure 2.pdf]

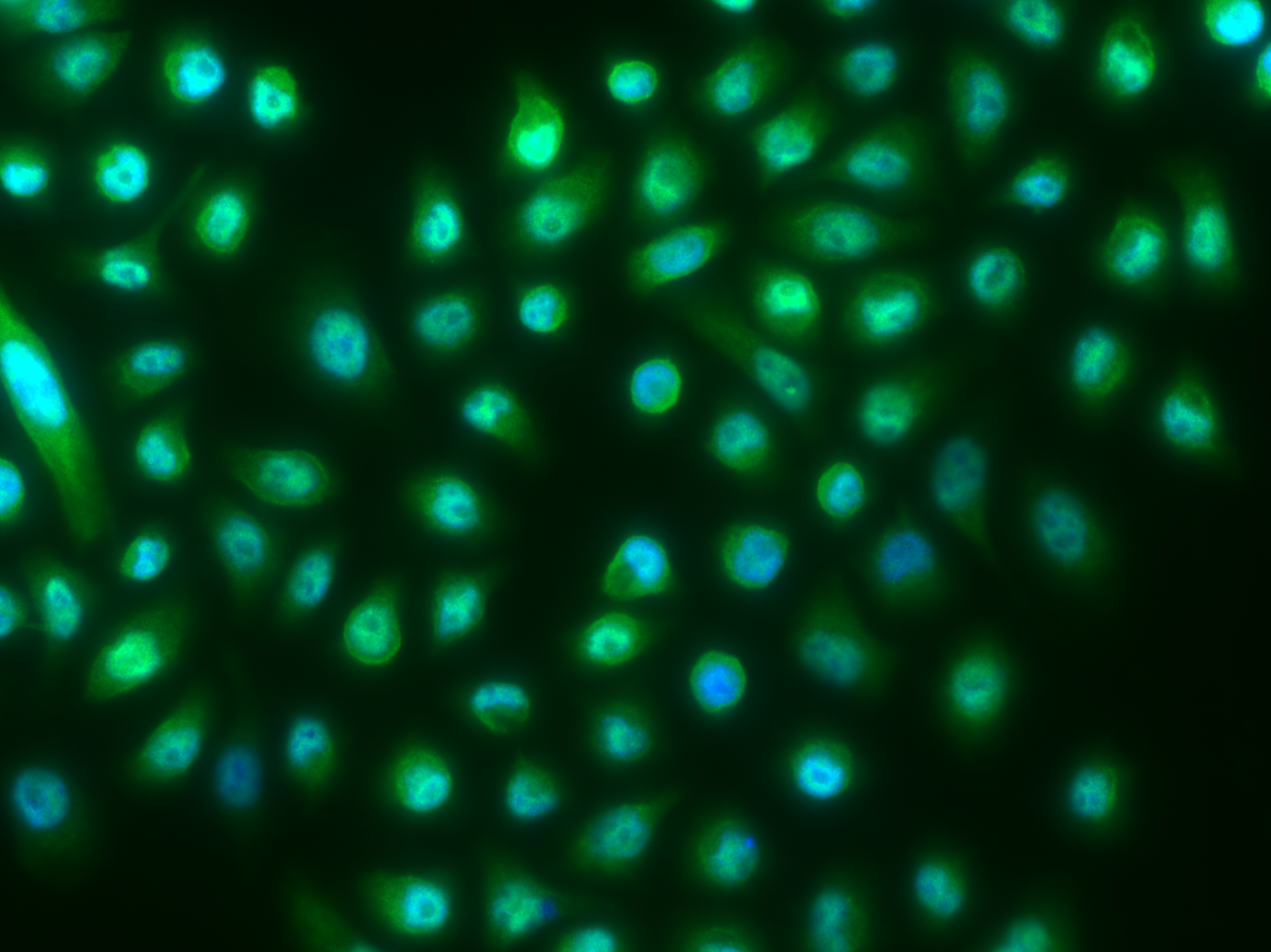

Supplement: Supplementary file 5 — Source Data Fig. 2 [file 44318_2024_40_MOESM5_ESM.zip › Figure 2/2I/2I RWPE-1 MM.TIF]

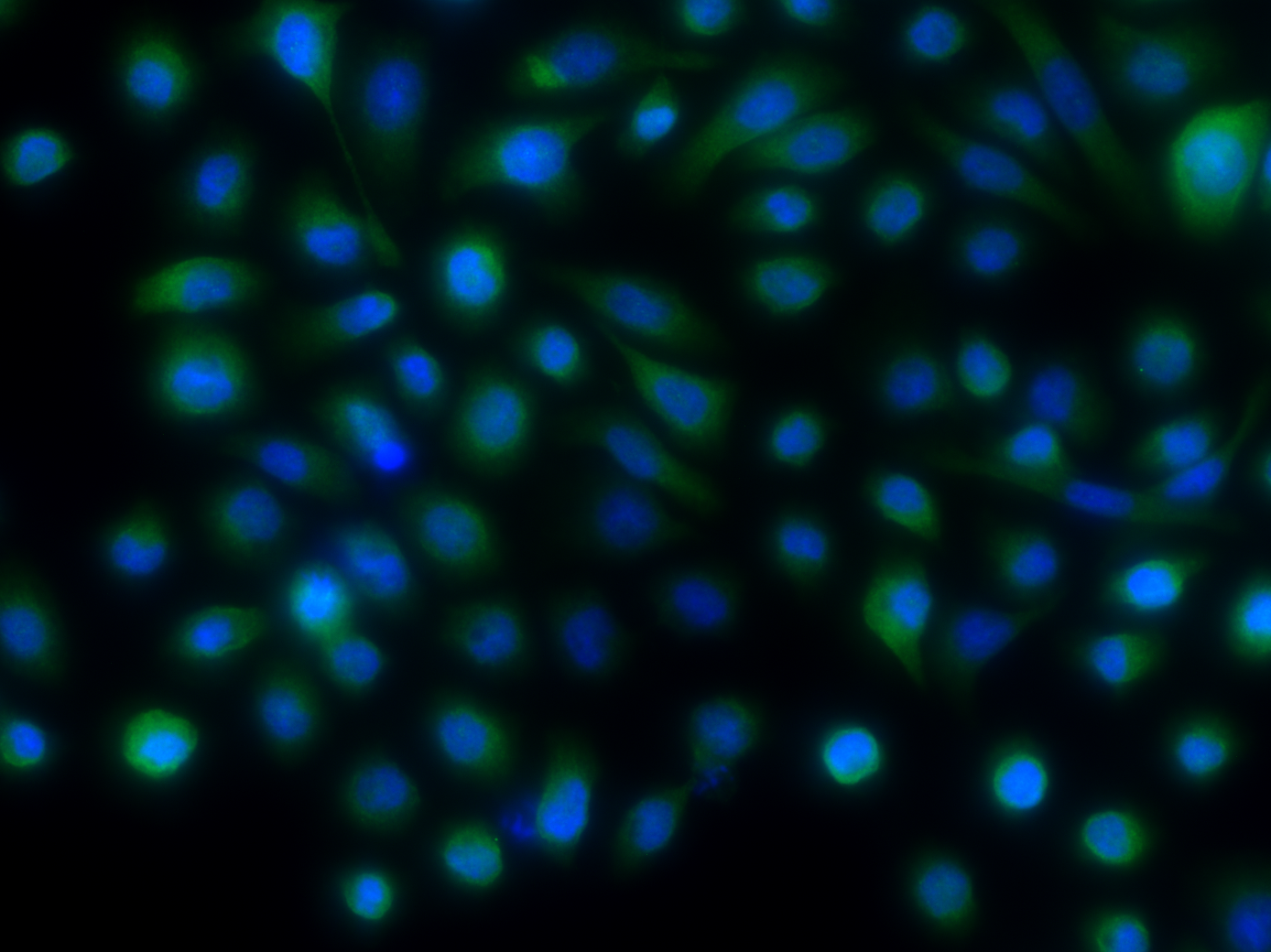

Supplement: Supplementary file 5 — Source Data Fig. 2 [file 44318_2024_40_MOESM5_ESM.zip › Figure 2/2I/2I RWPE WT.TIF]

Fig 3D

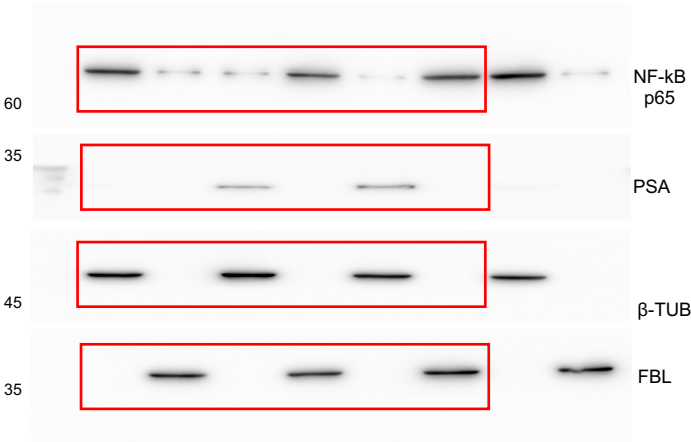

Fig 3F

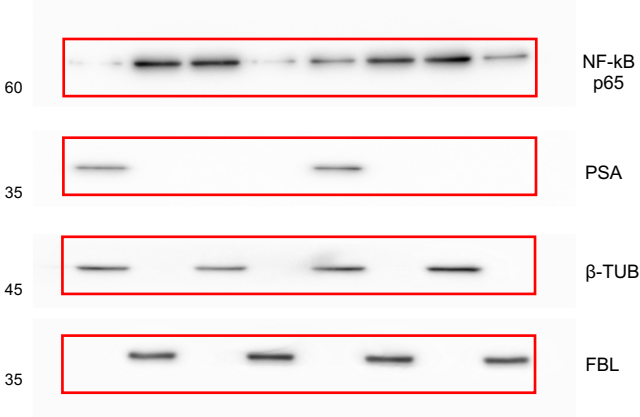

Fig 3

NF-kB p65  
PSA  
β-TUB  
FBL

Fig 3E

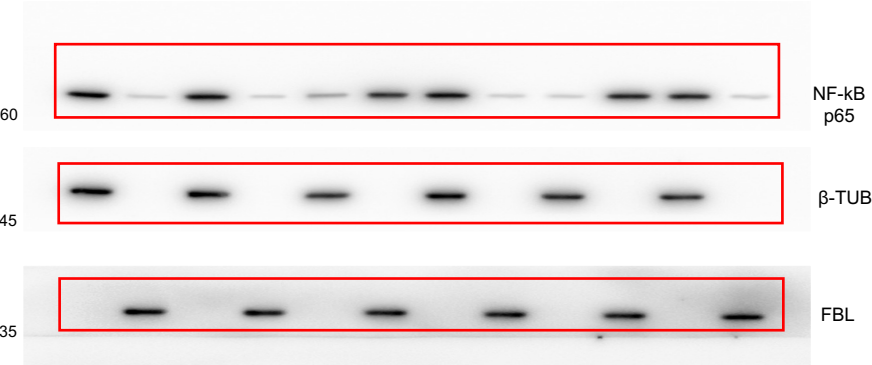

Fig 3H

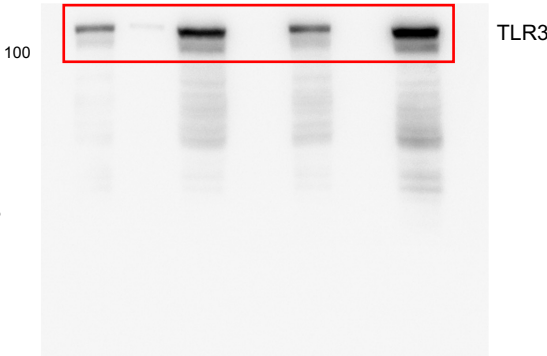

Fig 3G

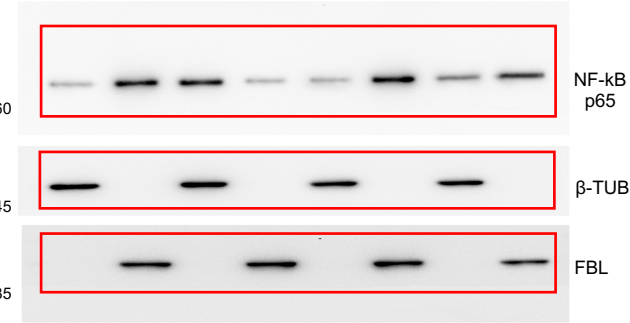

Fig 3I

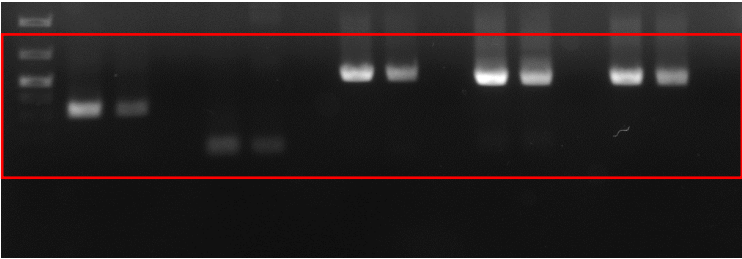

Fig 3J

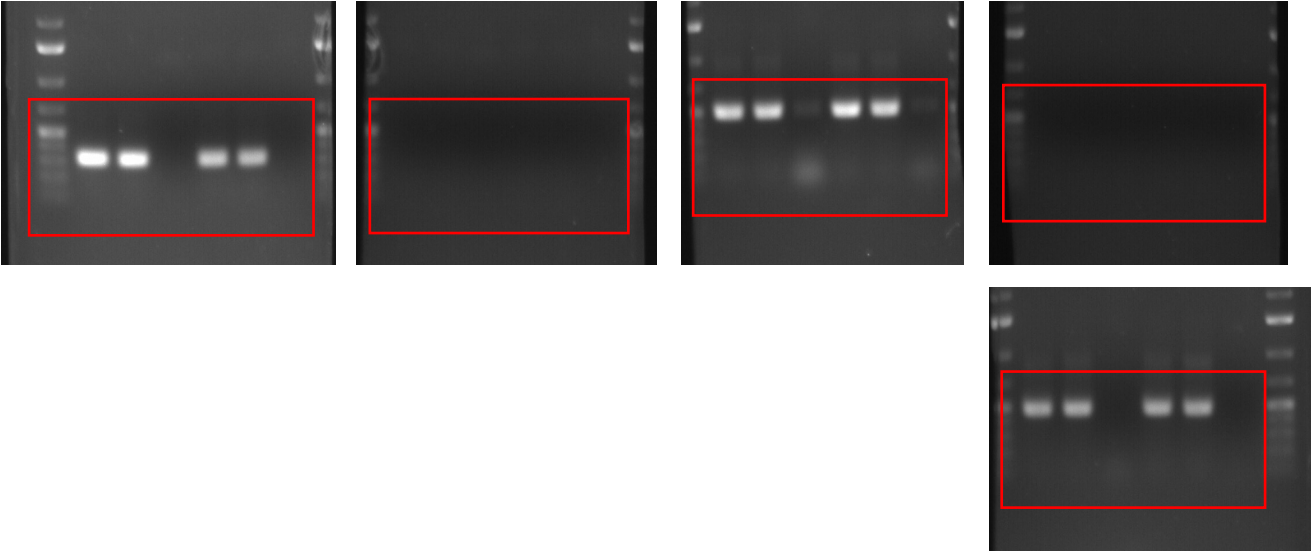

Supplement: Supplementary file 6 — Source Data Fig. 3 [file 44318_2024_40_MOESM6_ESM.zip › Figure 3/Raw Uncropped WB Figure 3.pdf]

Fig 4C

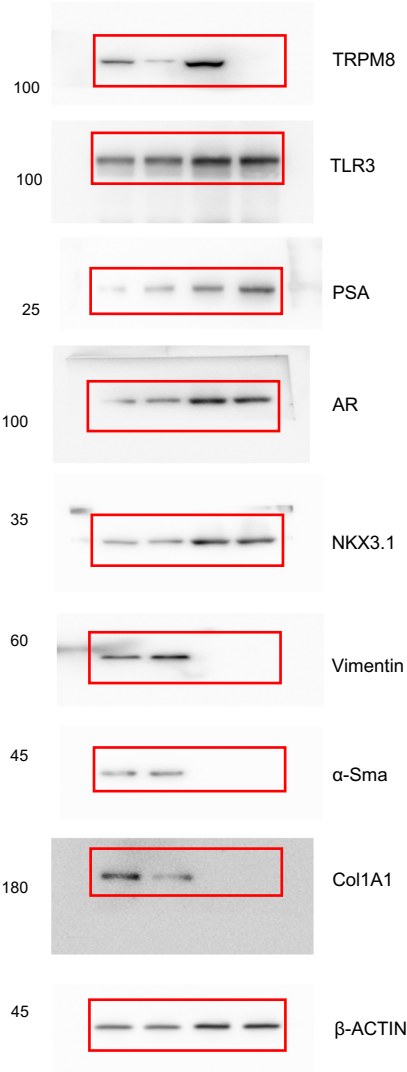

Fig 4F

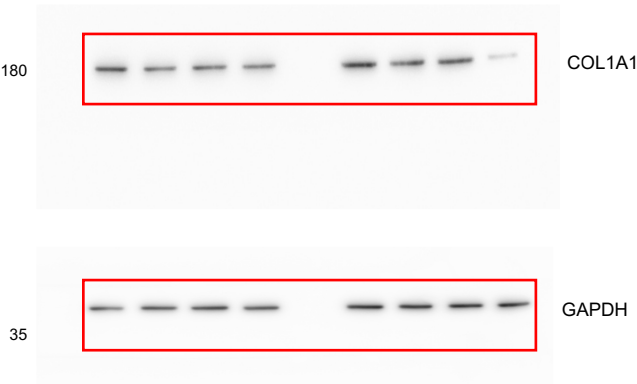

Supplement: Supplementary file 7 — Source Data Fig. 4 [file 44318_2024_40_MOESM7_ESM.zip › Figure 4/Raw Uncropped WB Figure 4.pdf]

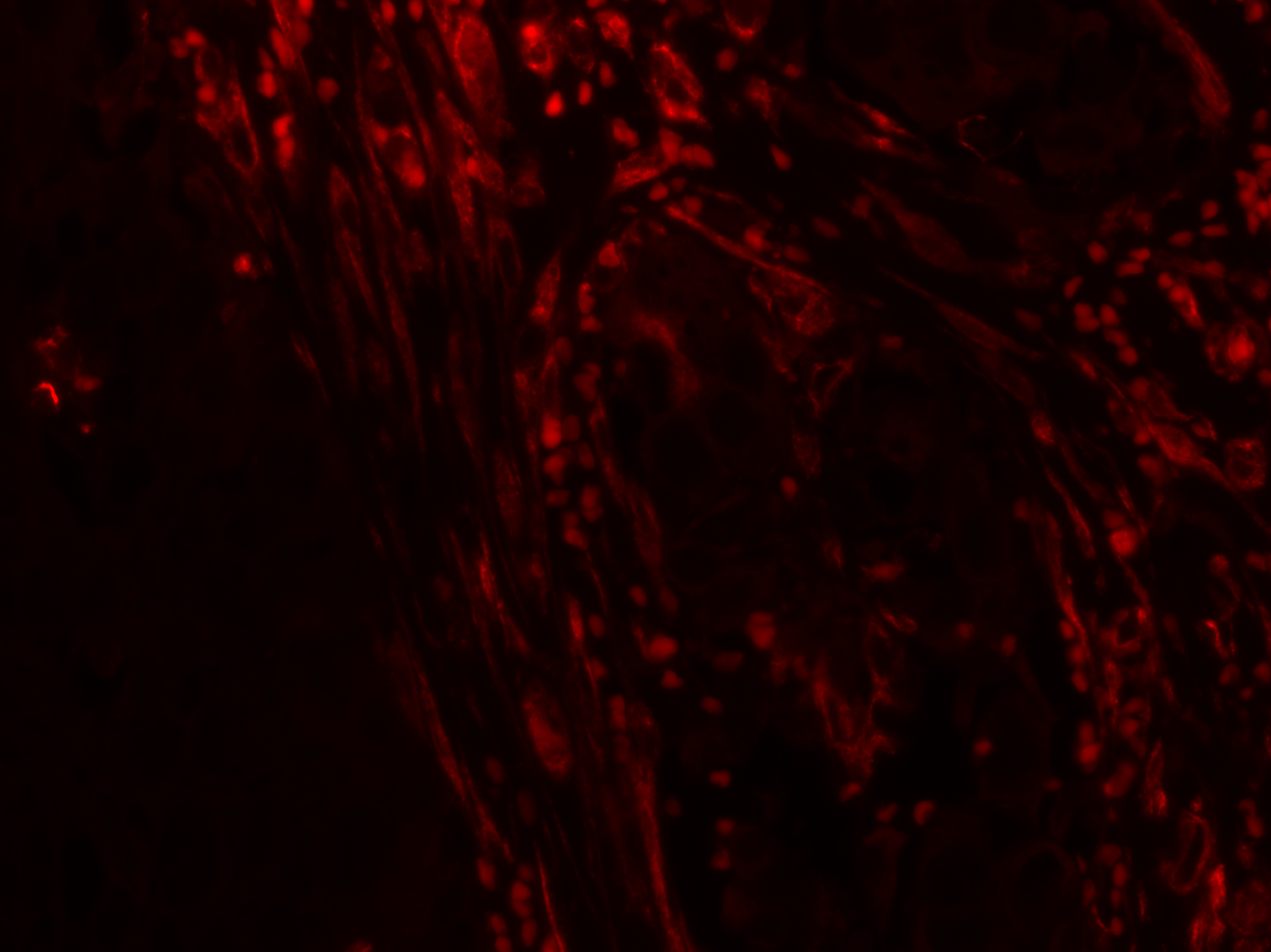

Supplement: Supplementary file 7 — Source Data Fig. 4 [file 44318_2024_40_MOESM7_ESM.zip › Figure 4/4E/4E Vim_r SMA_v WT 40x_c2.TIF]

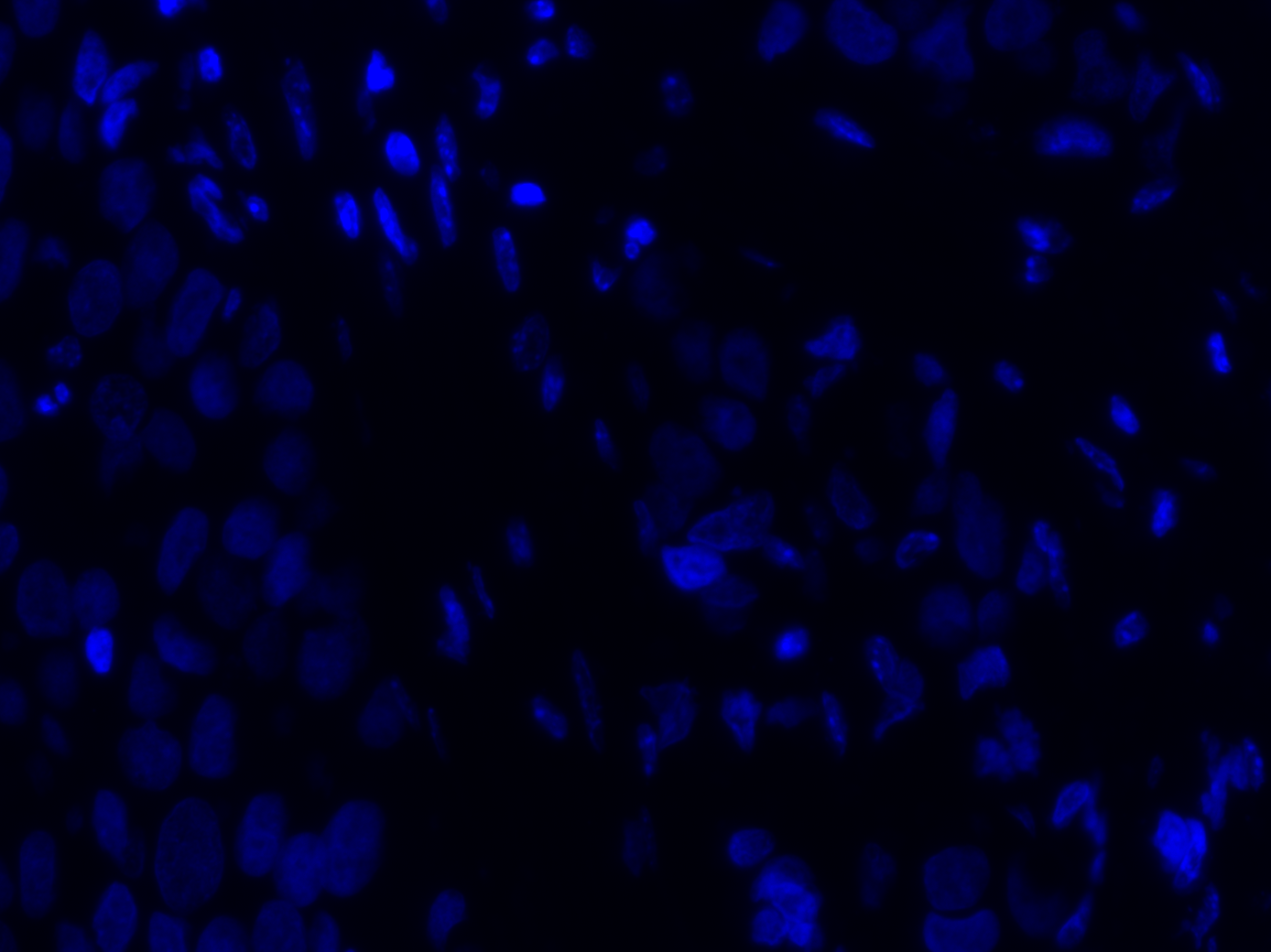

Supplement: Supplementary file 7 — Source Data Fig. 4 [file 44318_2024_40_MOESM7_ESM.zip › Figure 4/4E/4E Vim_r SMA_v WT 40x_c3.TIF]

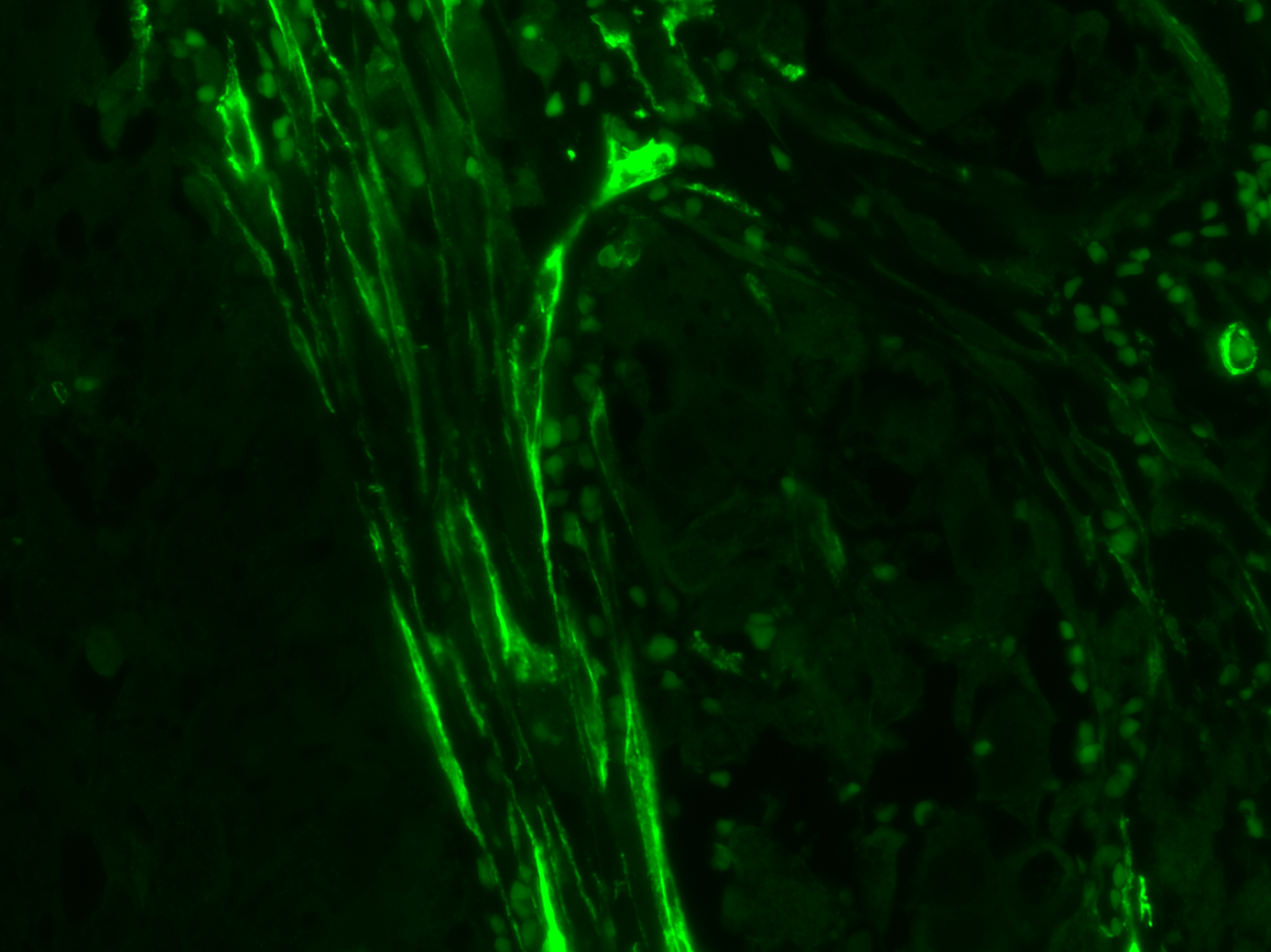

Supplement: Supplementary file 7 — Source Data Fig. 4 [file 44318_2024_40_MOESM7_ESM.zip › Figure 4/4E/4E Vim_r SMA_v WT 40x_c1.TIF]

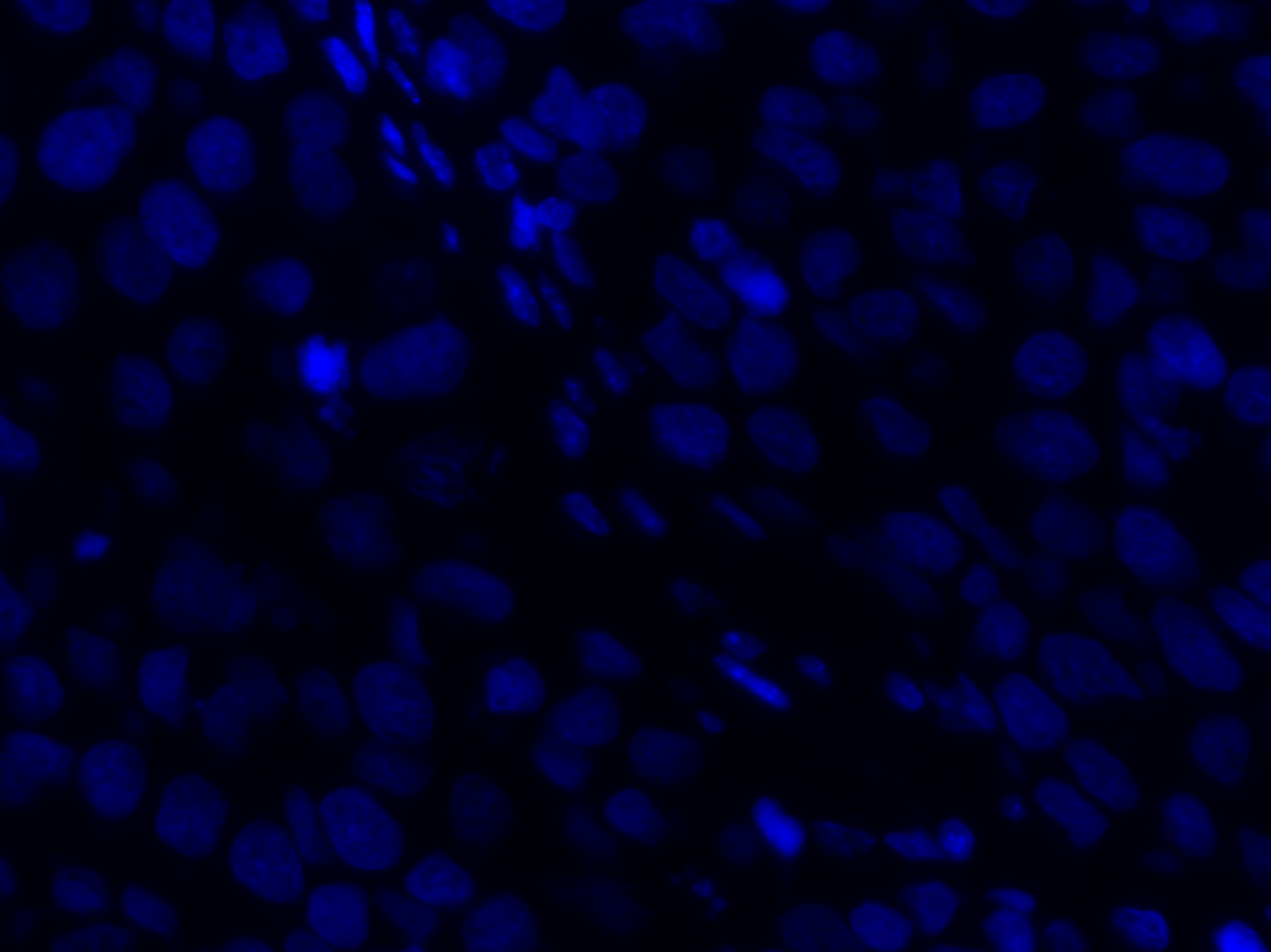

Supplement: Supplementary file 7 — Source Data Fig. 4 [file 44318_2024_40_MOESM7_ESM.zip › Figure 4/4E/4E Vim_r SMA_v MM 40x_c3.TIF]

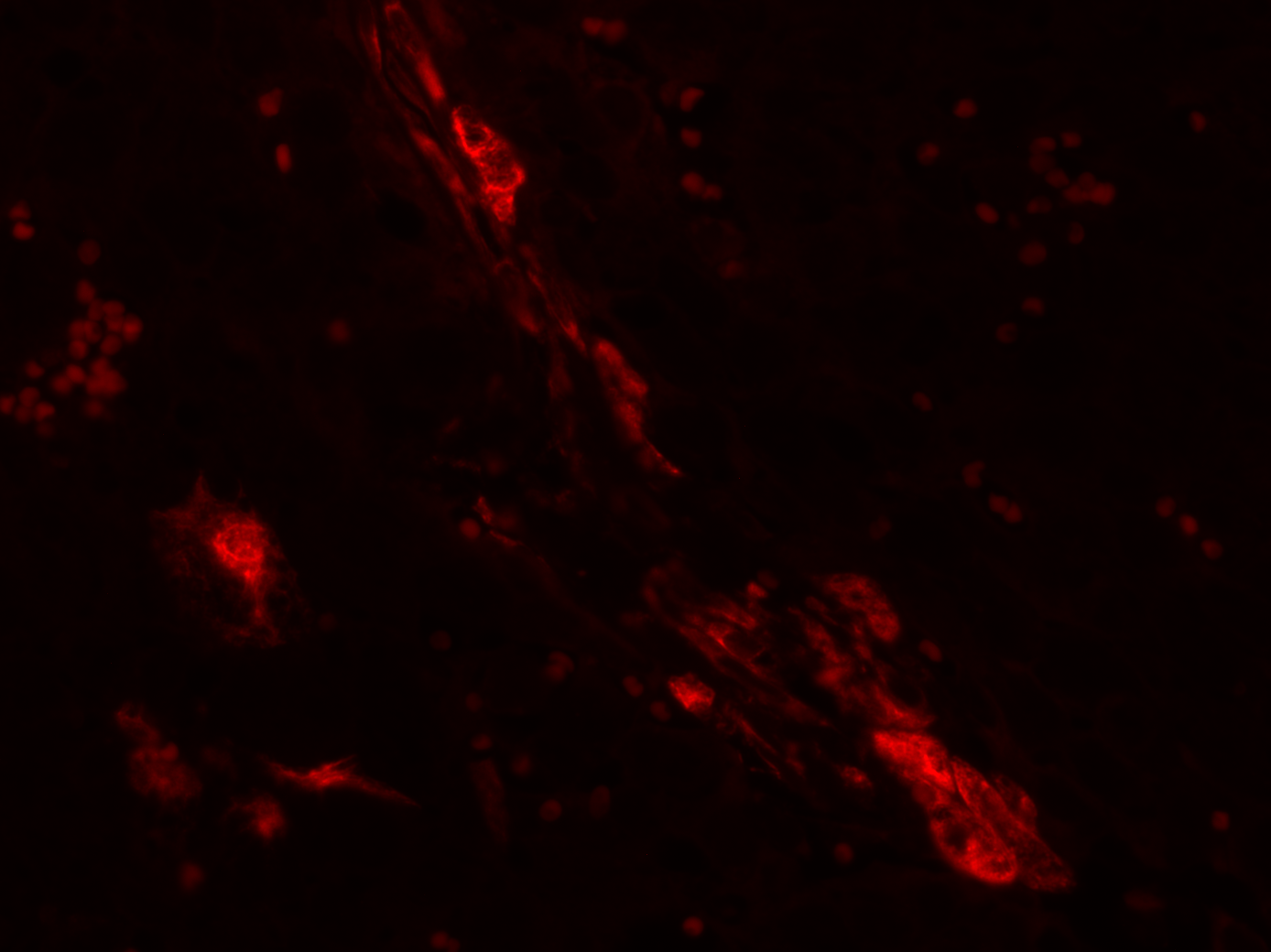

Supplement: Supplementary file 7 — Source Data Fig. 4 [file 44318_2024_40_MOESM7_ESM.zip › Figure 4/4E/4E Vim_r SMA_v MM 40x_c2.TIF]

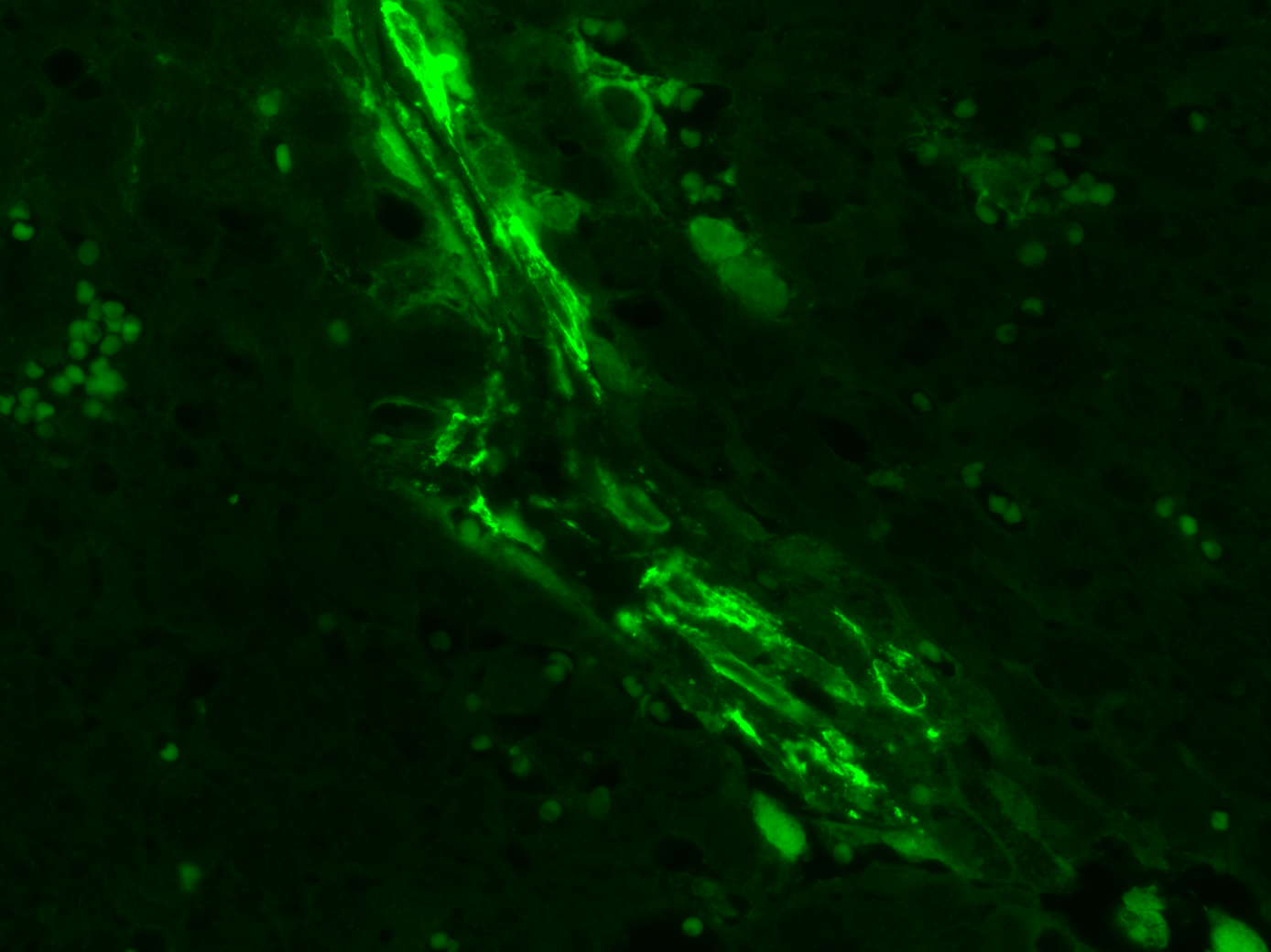

Supplement: Supplementary file 7 — Source Data Fig. 4 [file 44318_2024_40_MOESM7_ESM.zip › Figure 4/4E/4E Vim_r SMA_v MM 40x_c1.TIF]

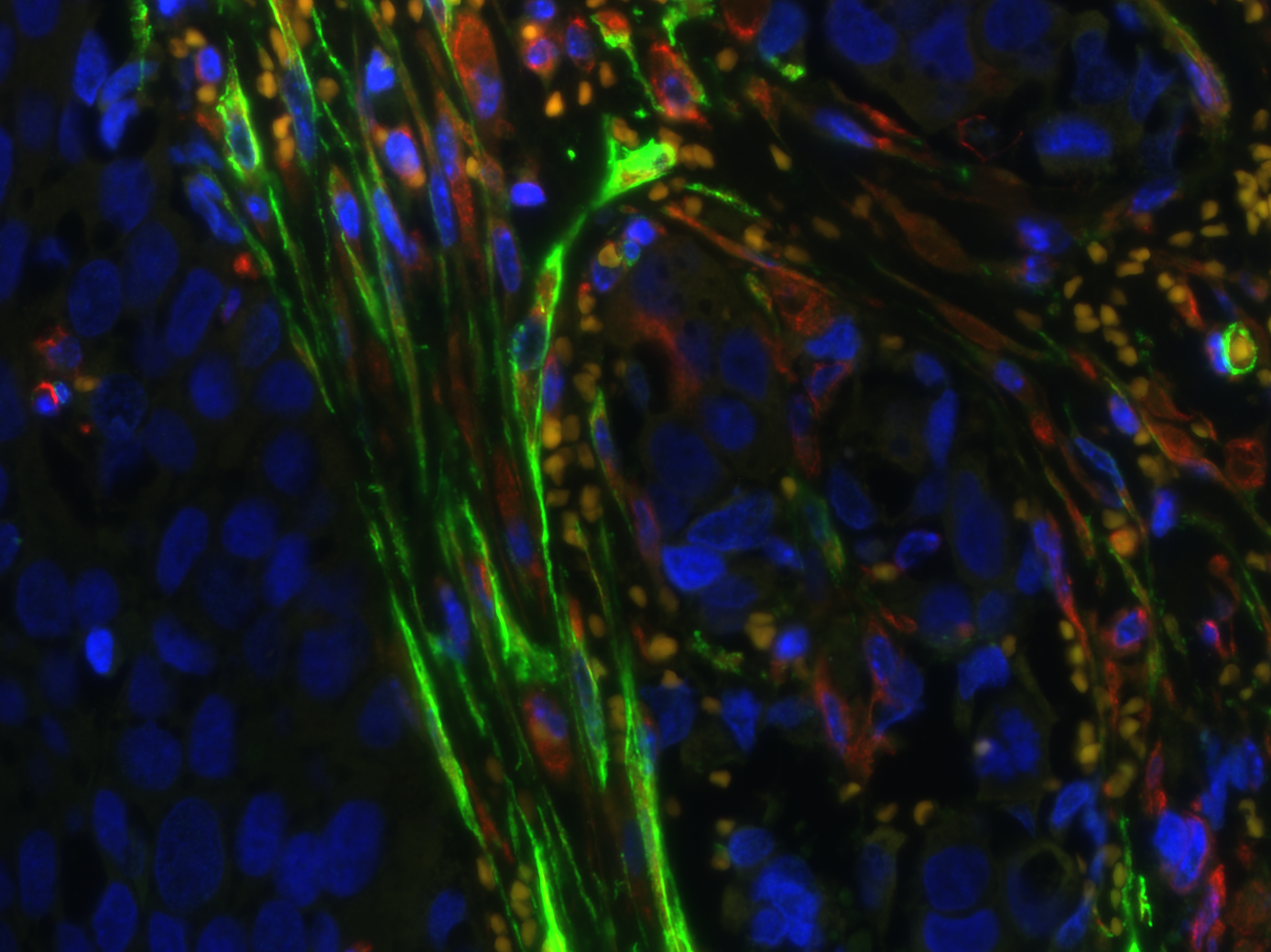

Supplement: Supplementary file 7 — Source Data Fig. 4 [file 44318_2024_40_MOESM7_ESM.zip › Figure 4/4E/4E Vim_r SMA_v WT 40x_(c1+c2+c3).TIF]

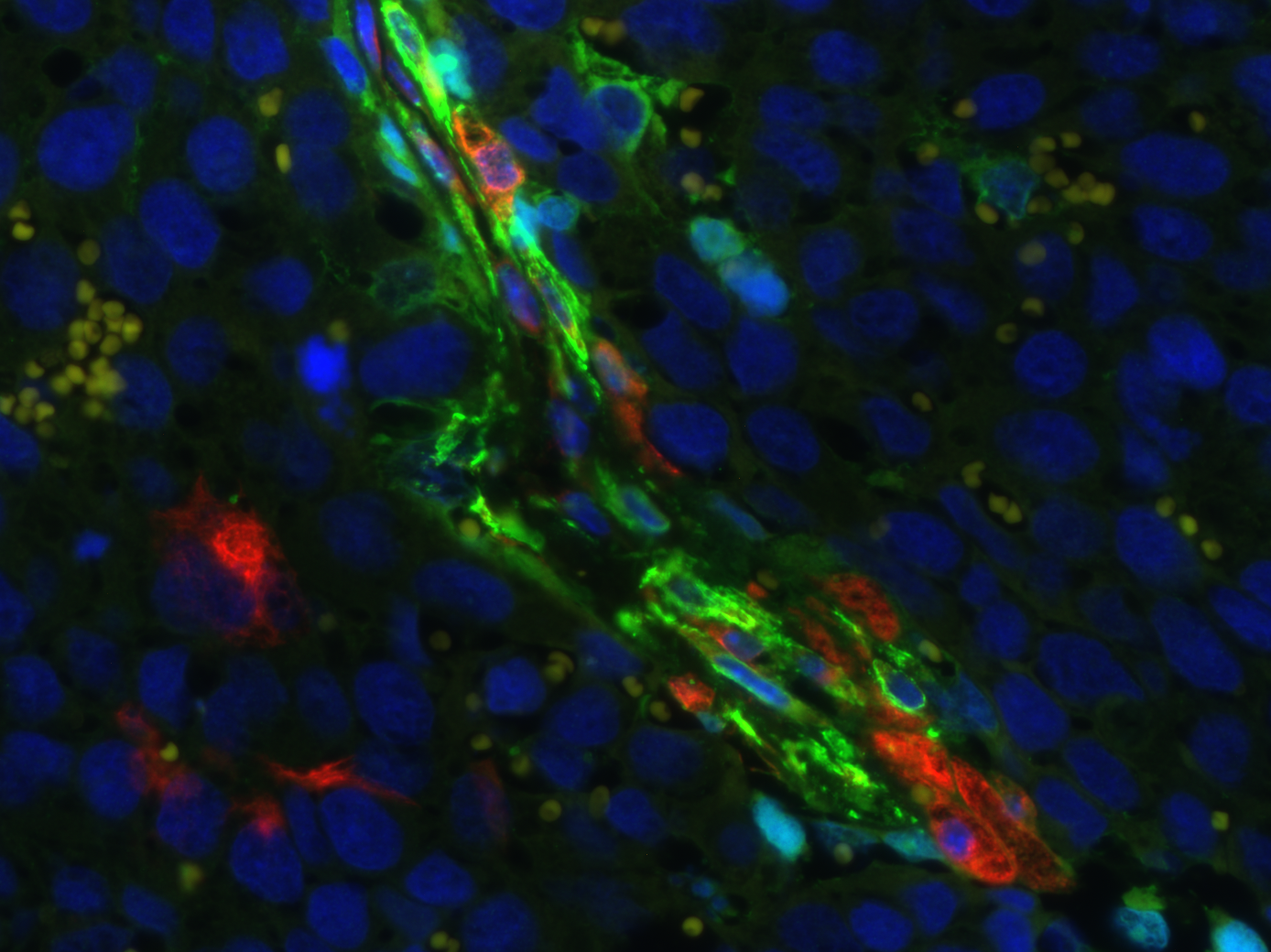

Supplement: Supplementary file 7 — Source Data Fig. 4 [file 44318_2024_40_MOESM7_ESM.zip › Figure 4/4E/4E Vim_r SMA_v MM 40x_(c1+c2+c3).TIF]

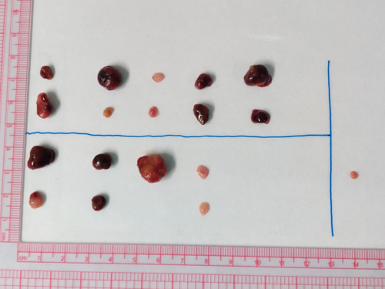

Supplement: Supplementary file 7 — Source Data Fig. 4 [file 44318_2024_40_MOESM7_ESM.zip › Figure 4/4B/4B.png]

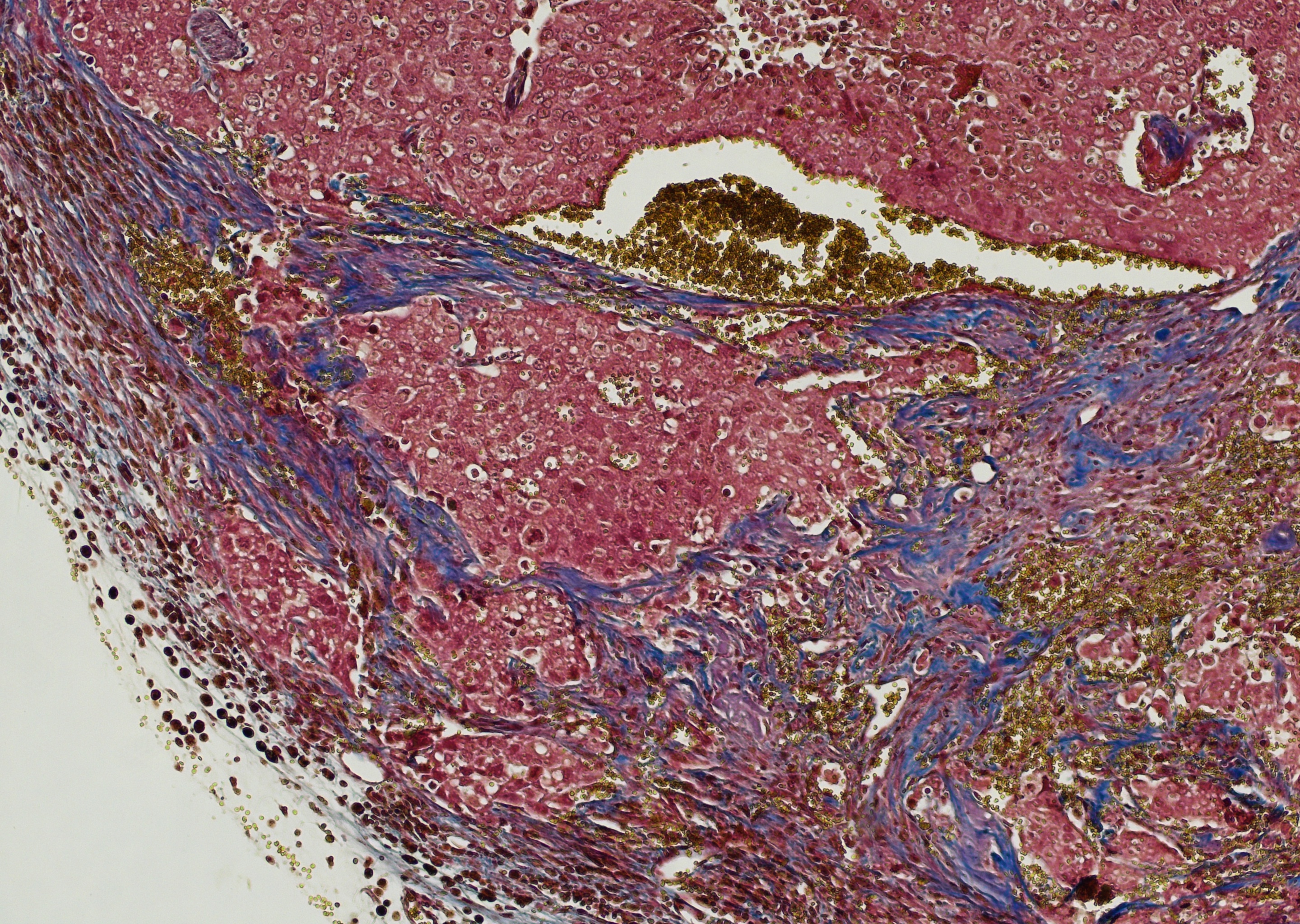

Supplement: Supplementary file 7 — Source Data Fig. 4 [file 44318_2024_40_MOESM7_ESM.zip › Figure 4/4D/4D WT.jpg]

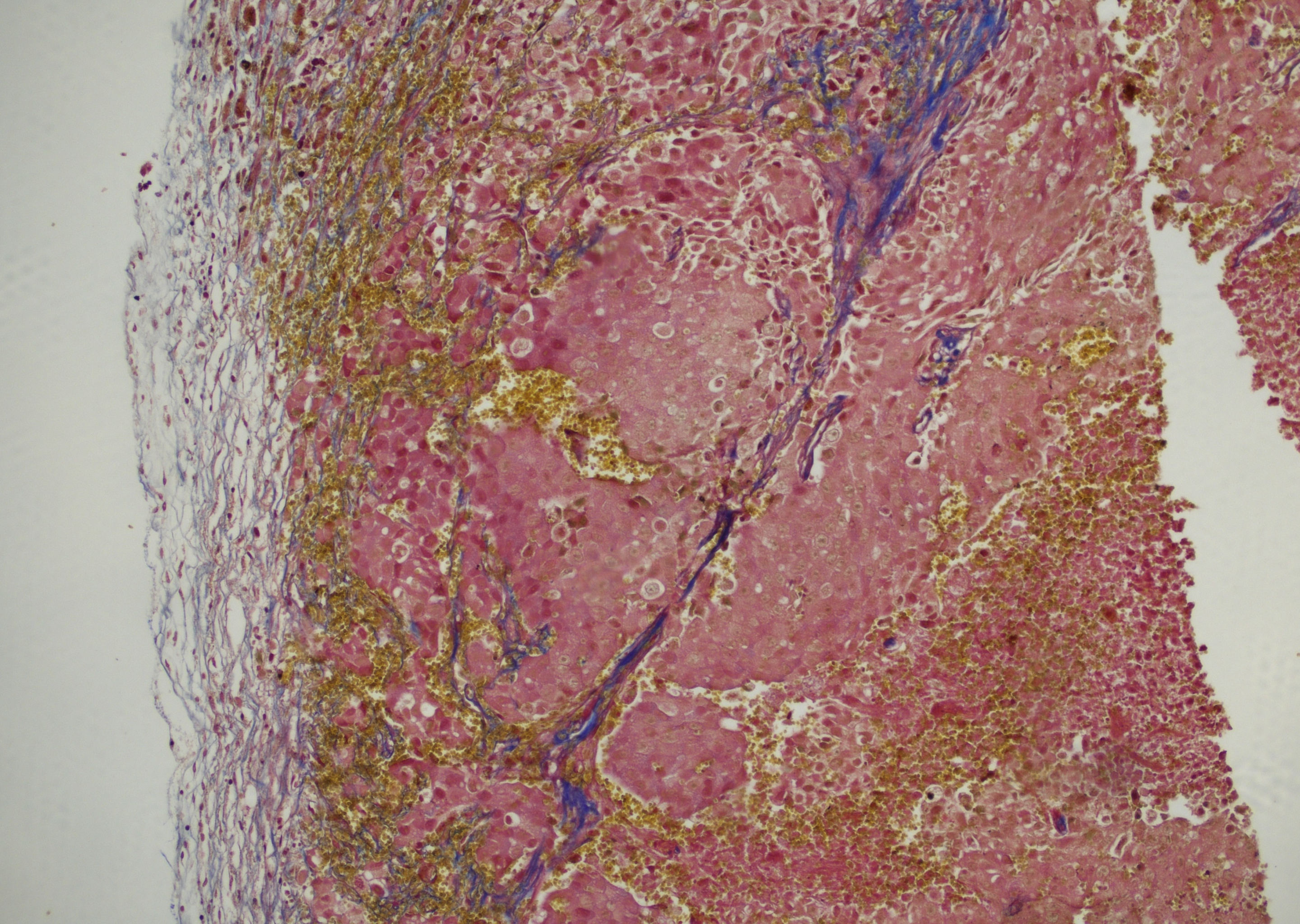

Supplement: Supplementary file 7 — Source Data Fig. 4 [file 44318_2024_40_MOESM7_ESM.zip › Figure 4/4D/4D MM.jpg]

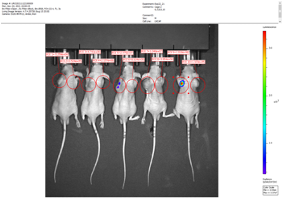

Supplement: Supplementary file 7 — Source Data Fig. 4 [file 44318_2024_40_MOESM7_ESM.zip › Figure 4/4A/4A WT.png]

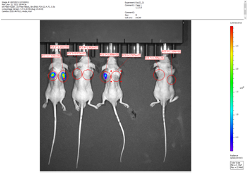

Supplement: Supplementary file 7 — Source Data Fig. 4 [file 44318_2024_40_MOESM7_ESM.zip › Figure 4/4A/4A MM.png]

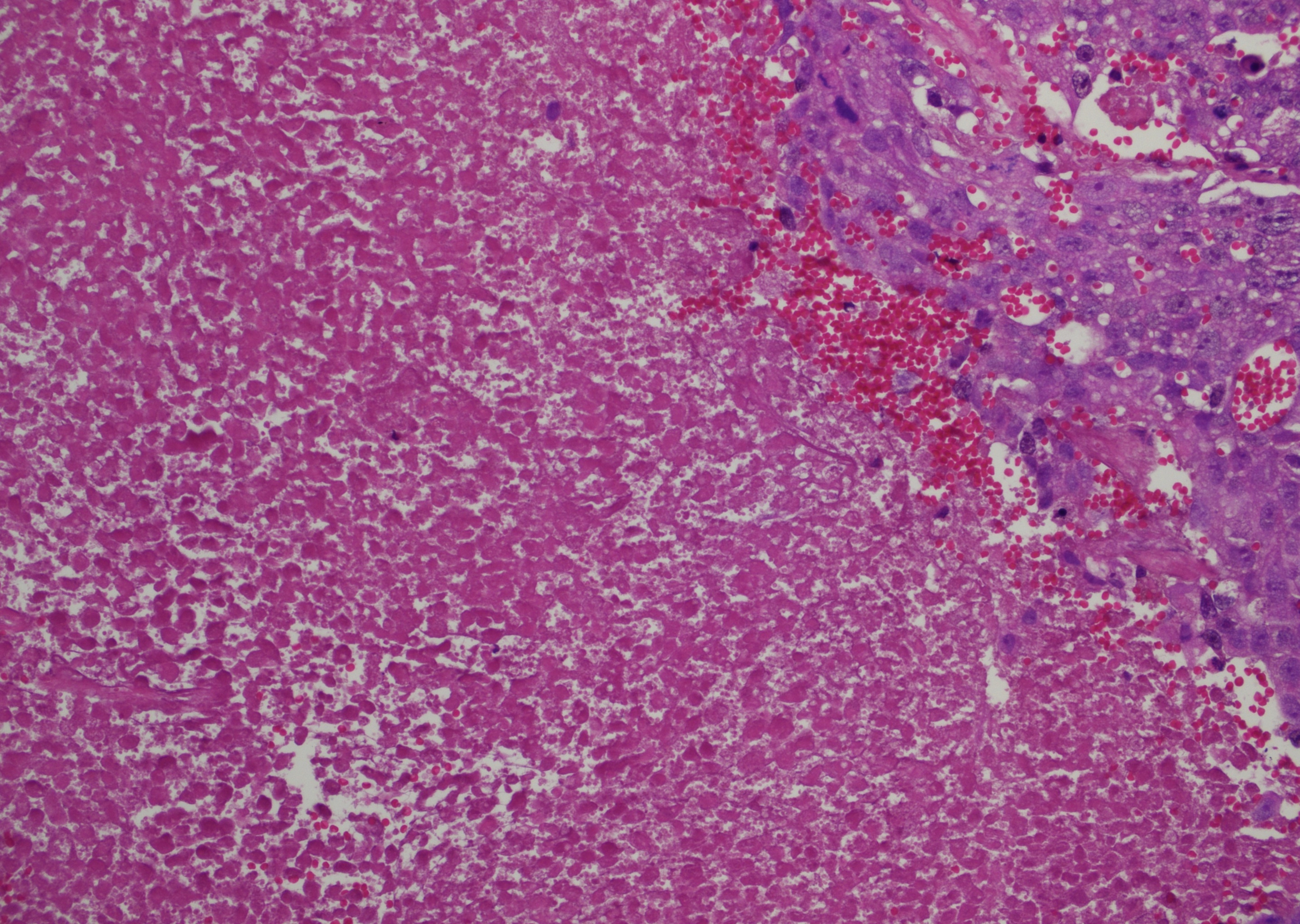

Supplement: Supplementary file 7 — Source Data Fig. 4 [file 44318_2024_40_MOESM7_ESM.zip › Figure 4/4H/4H NECROSIS MM 20x.jpg]

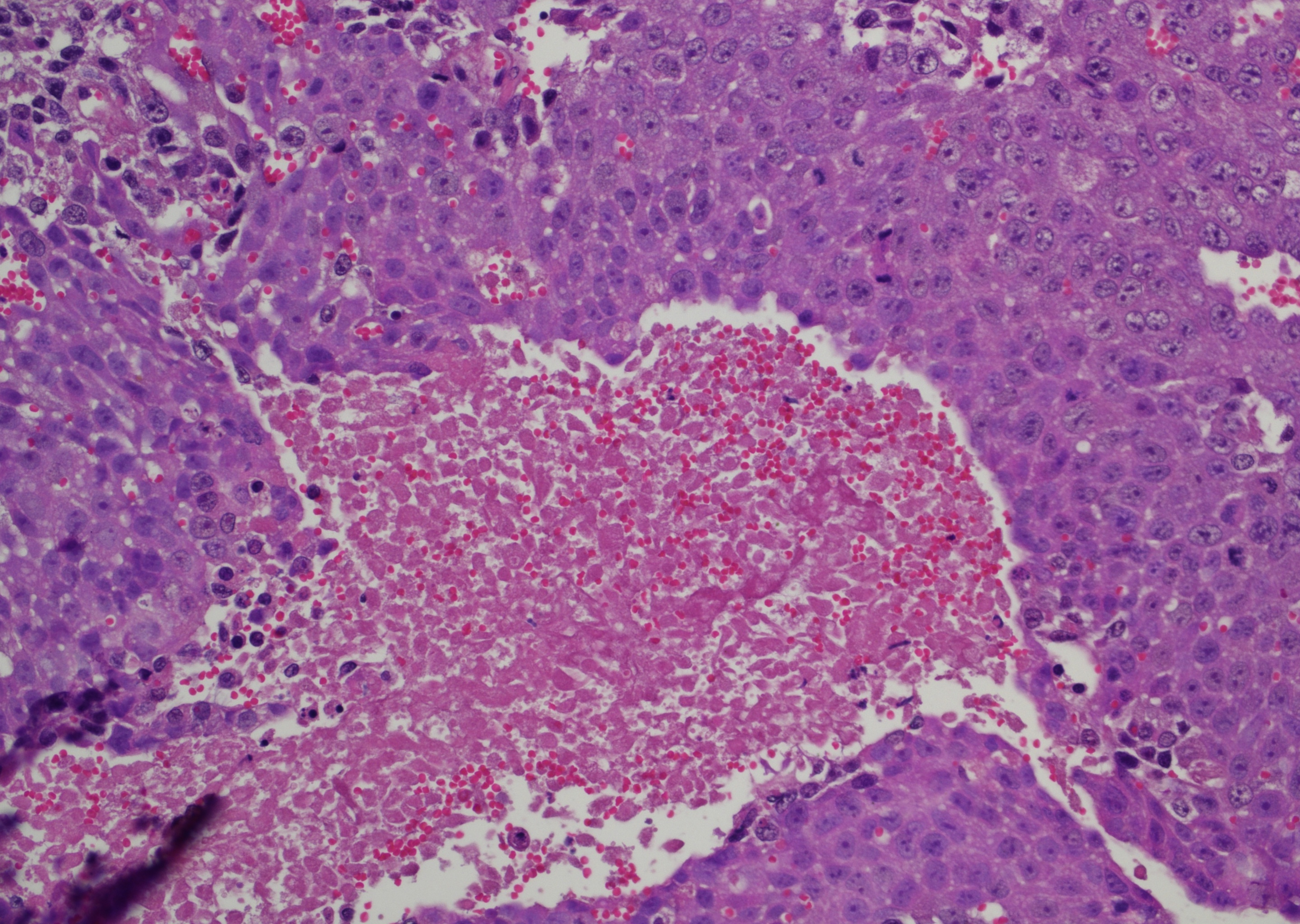

Supplement: Supplementary file 7 — Source Data Fig. 4 [file 44318_2024_40_MOESM7_ESM.zip › Figure 4/4H/4H NECROSIS WT 20x.jpg]

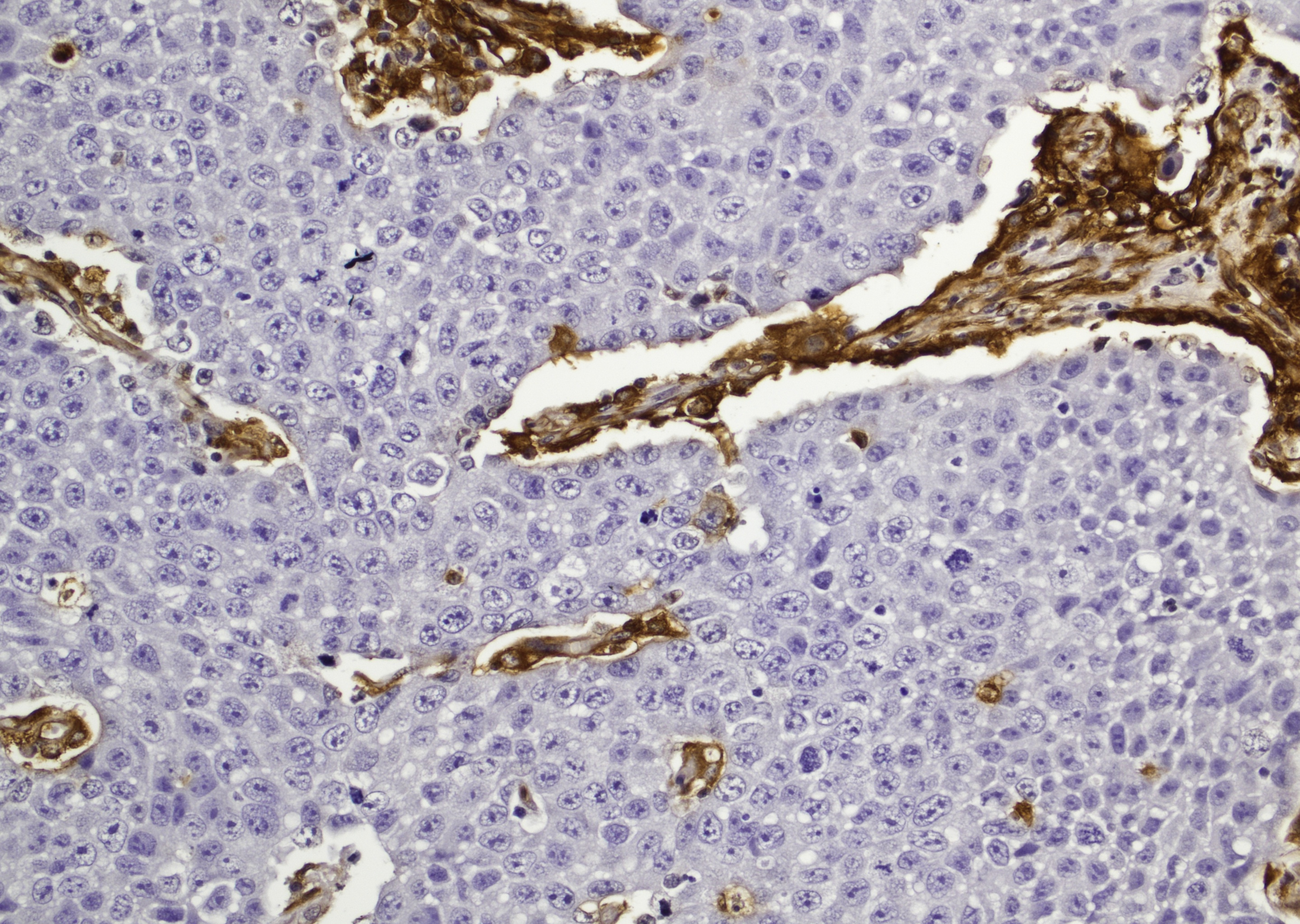

Supplement: Supplementary file 7 — Source Data Fig. 4 [file 44318_2024_40_MOESM7_ESM.zip › Figure 4/4H/4H MHC-II MM 20x.jpg]

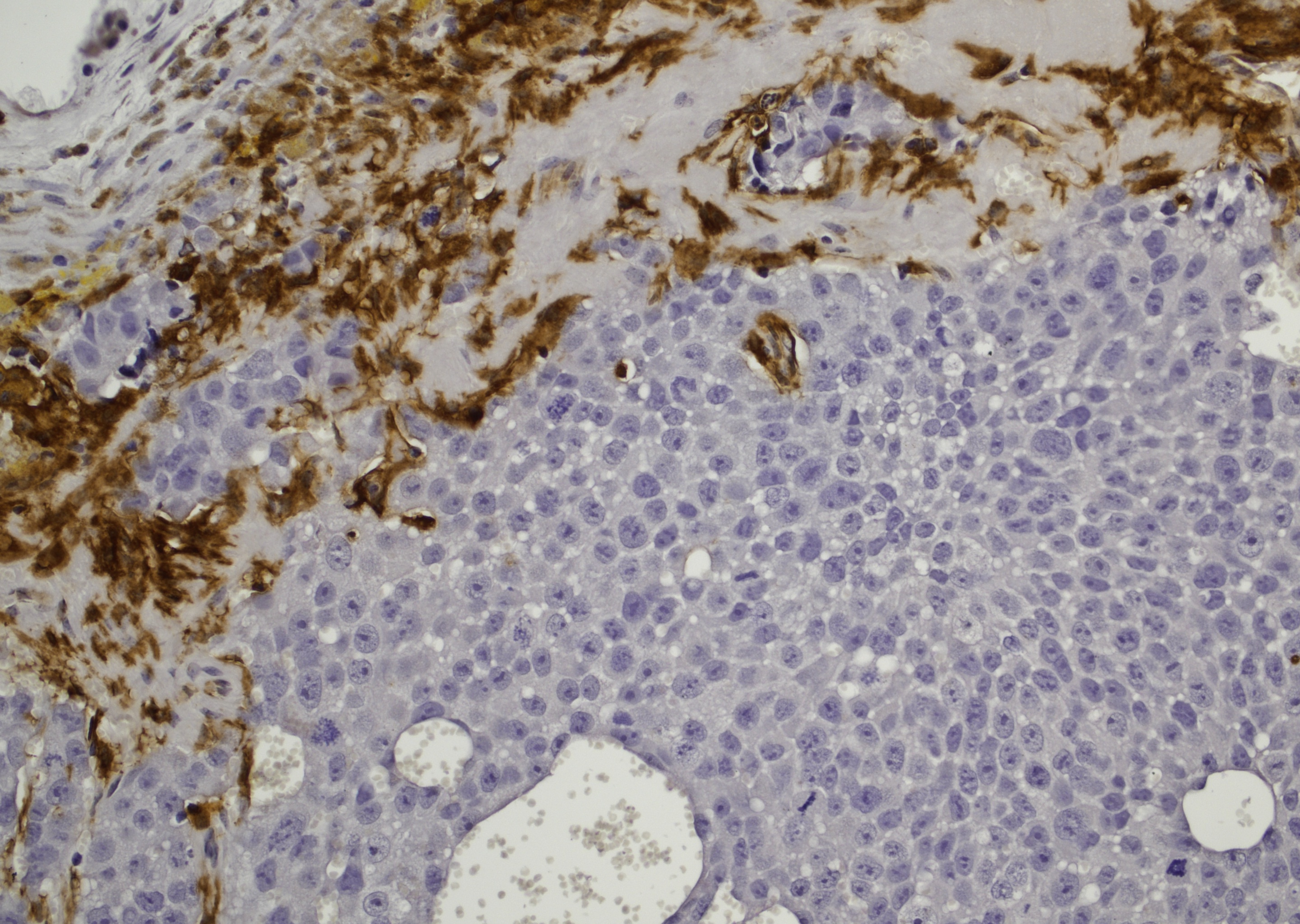

Supplement: Supplementary file 7 — Source Data Fig. 4 [file 44318_2024_40_MOESM7_ESM.zip › Figure 4/4H/4H MHC-II WT 20x.jpg]

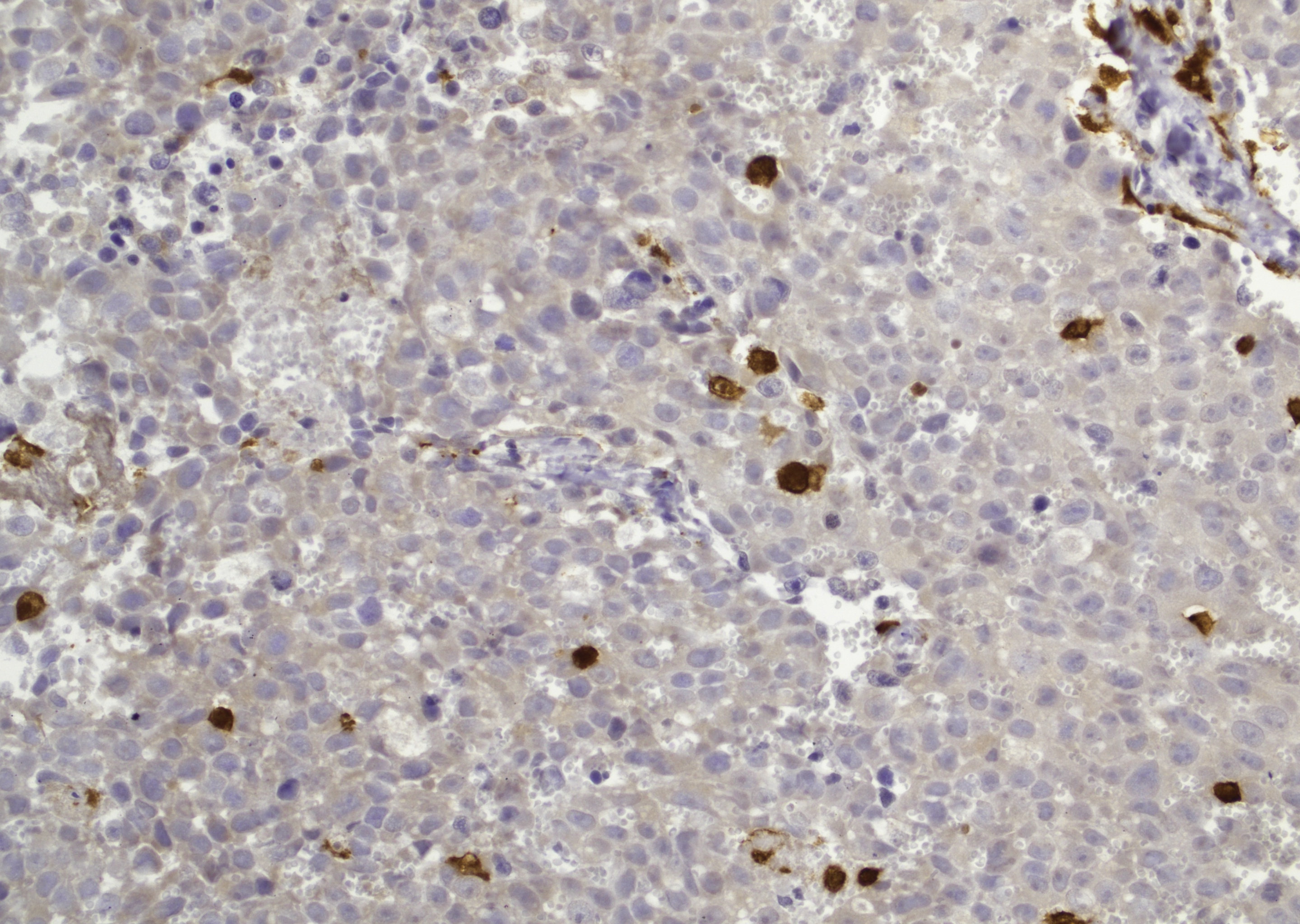

Supplement: Supplementary file 7 — Source Data Fig. 4 [file 44318_2024_40_MOESM7_ESM.zip › Figure 4/4H/4H IBA1 MM 20x.jpg]

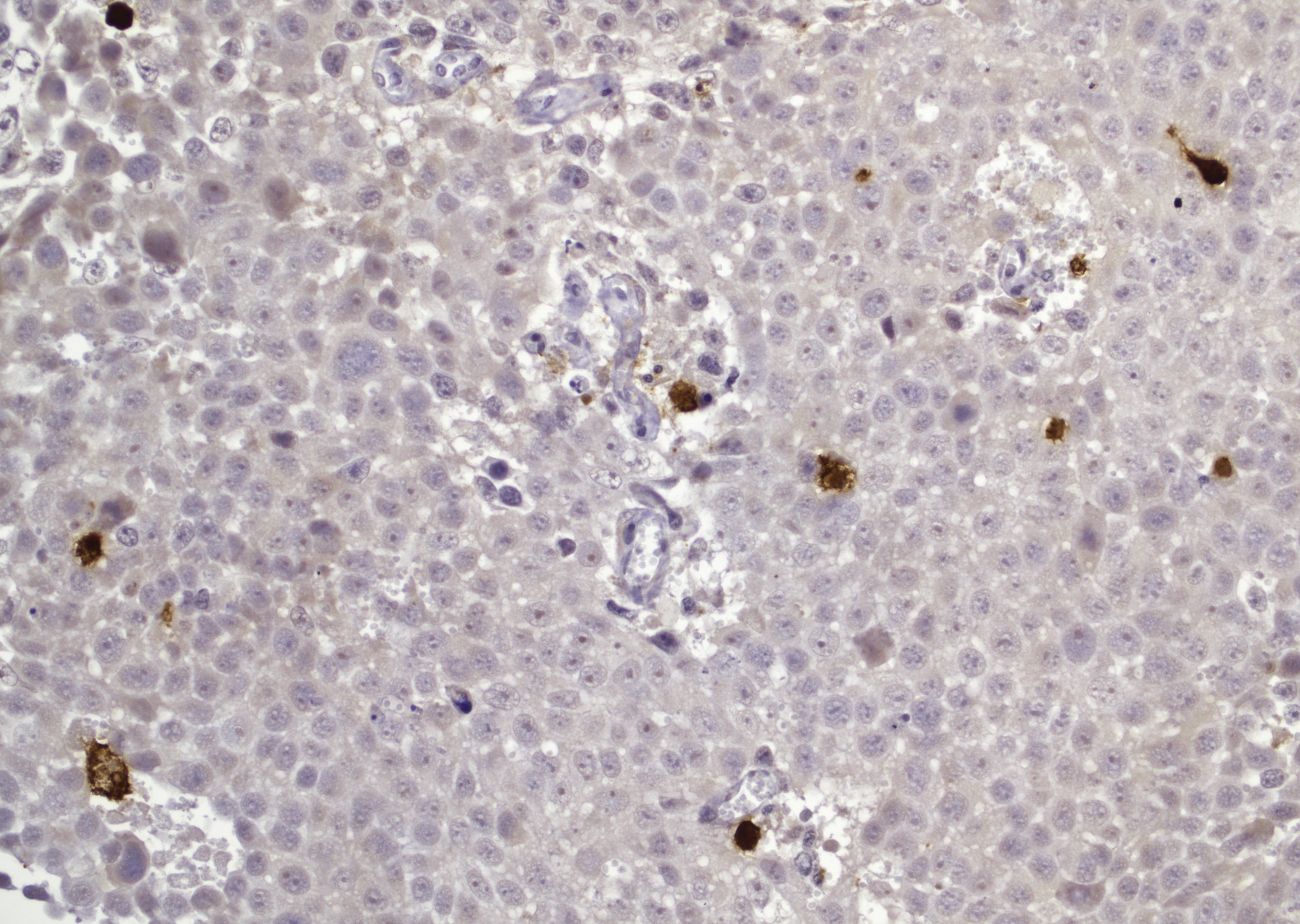

Supplement: Supplementary file 7 — Source Data Fig. 4 [file 44318_2024_40_MOESM7_ESM.zip › Figure 4/4H/4H IBA1 WT 20x.jpg]

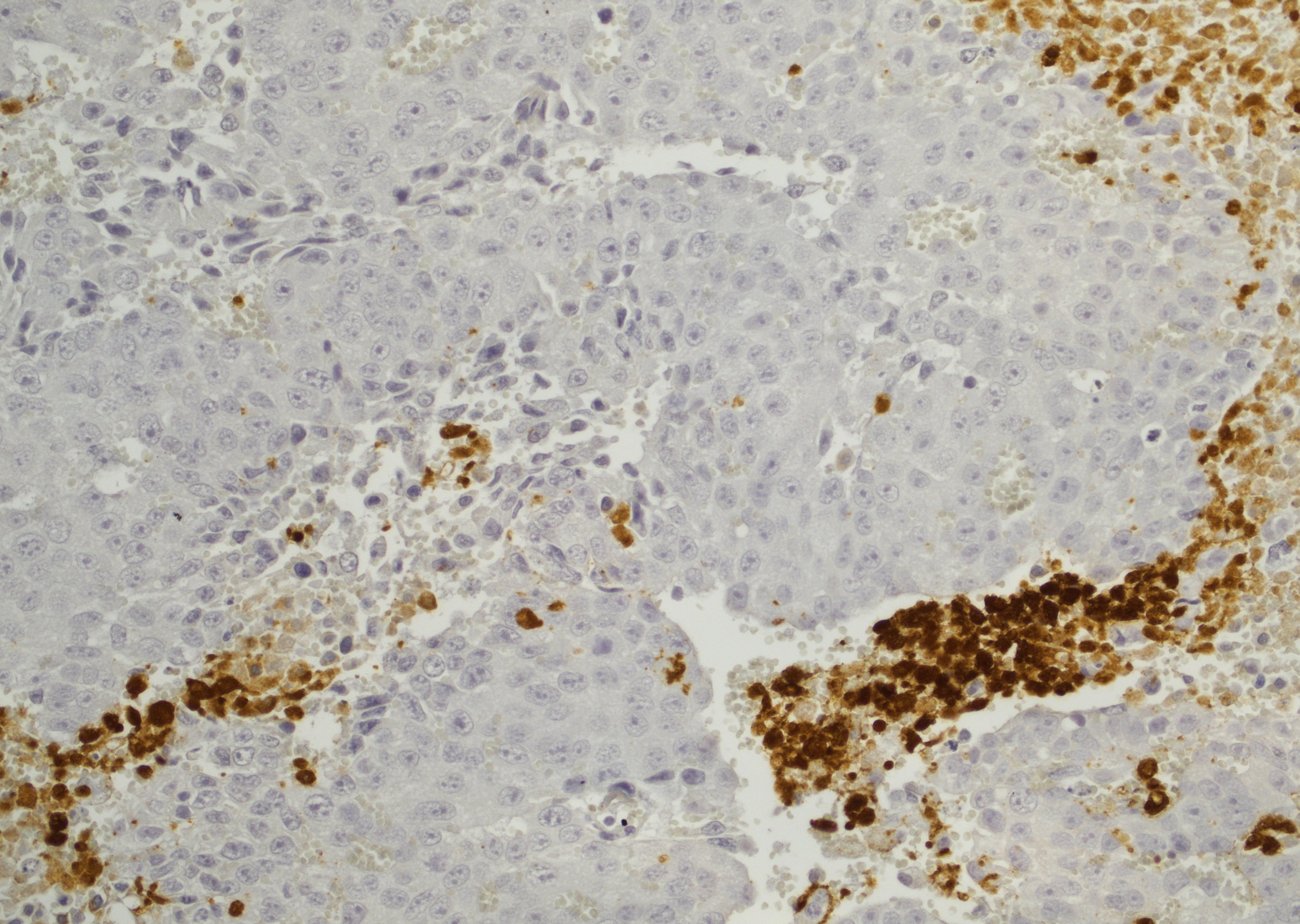

Supplement: Supplementary file 7 — Source Data Fig. 4 [file 44318_2024_40_MOESM7_ESM.zip › Figure 4/4H/4H NKp46 WT 20x.jpg]

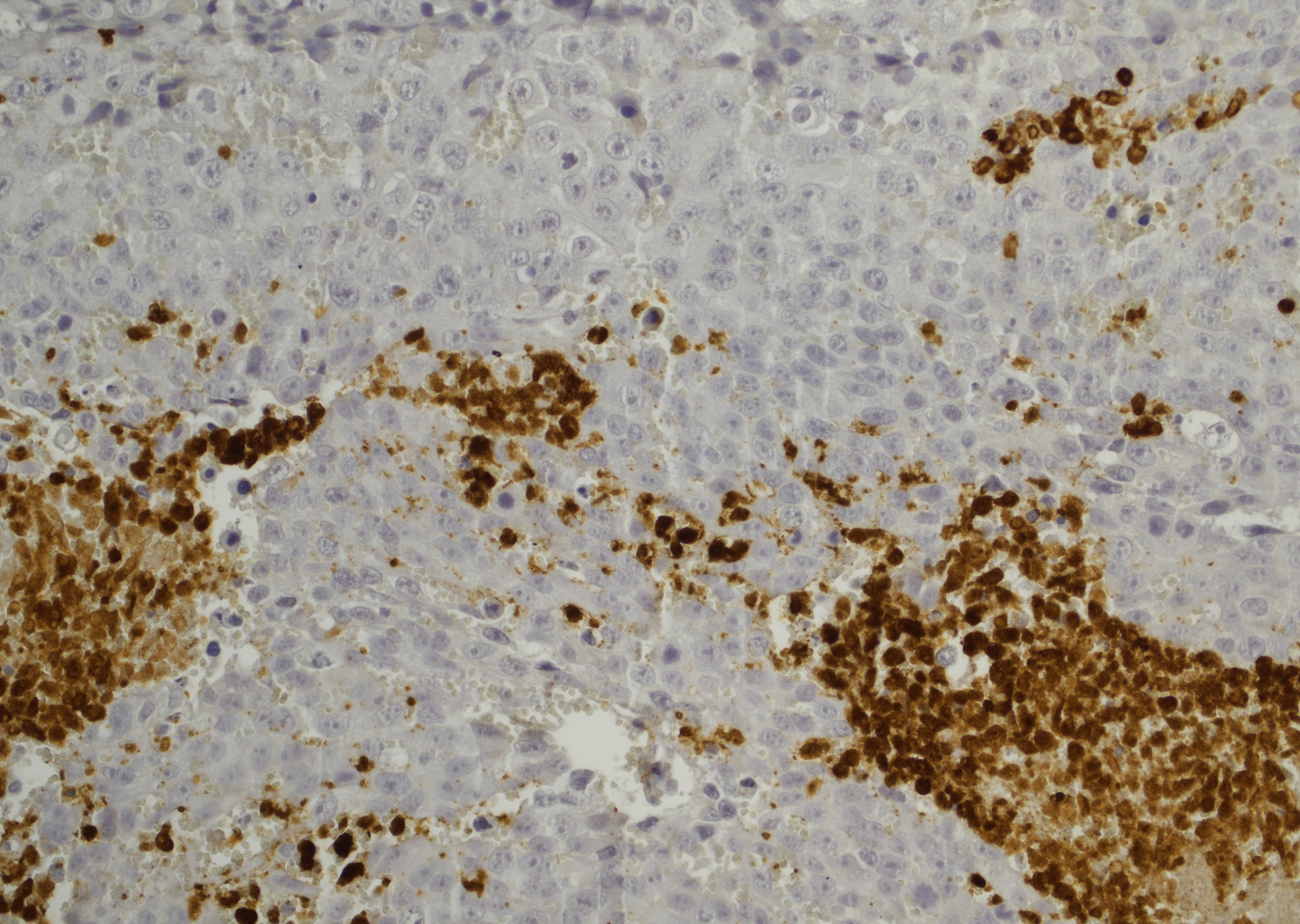

Supplement: Supplementary file 7 — Source Data Fig. 4 [file 44318_2024_40_MOESM7_ESM.zip › Figure 4/4H/4H NKp46 MM 20x.jpg]

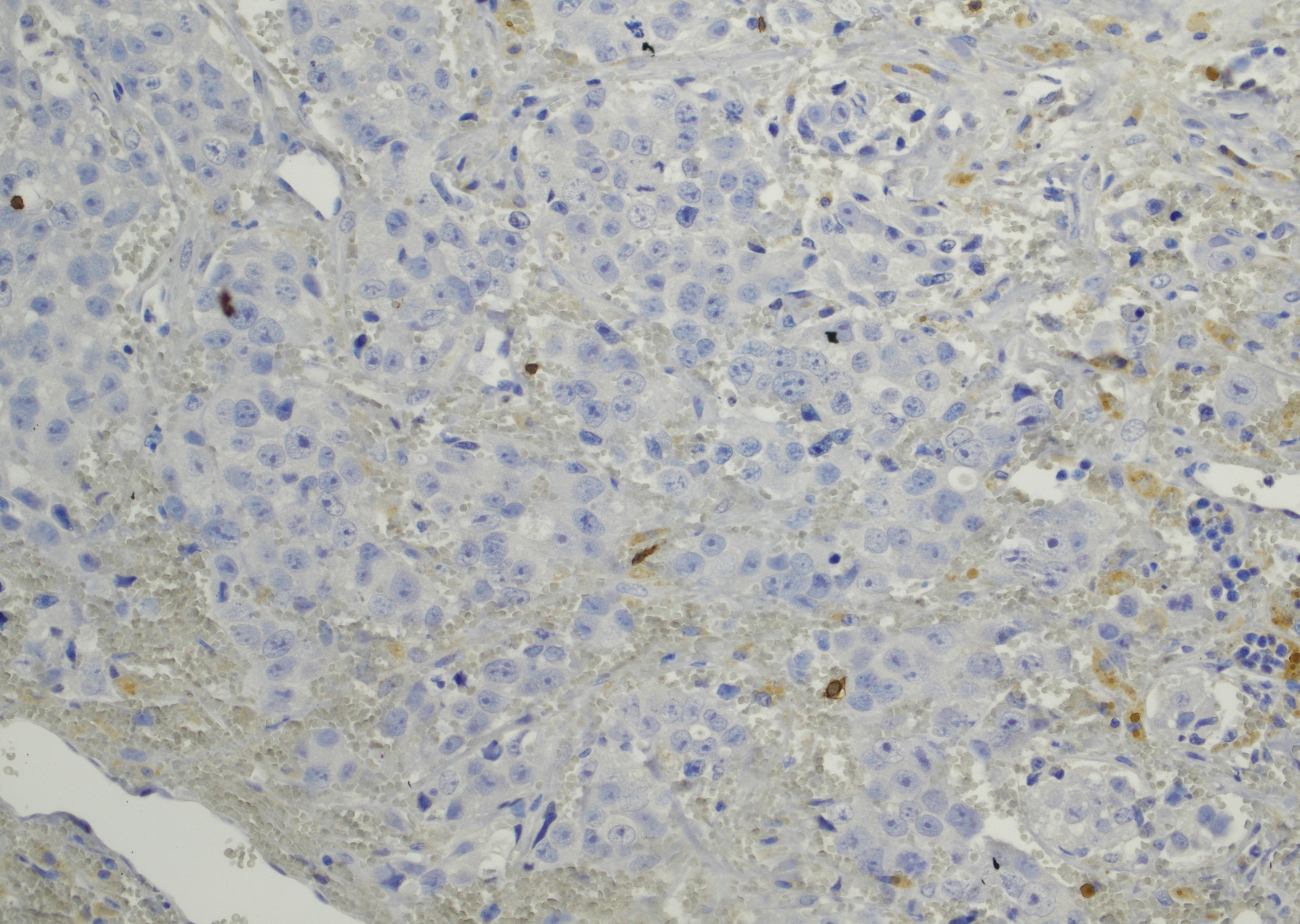

Supplement: Supplementary file 7 — Source Data Fig. 4 [file 44318_2024_40_MOESM7_ESM.zip › Figure 4/4H/4H B220 WT 20x.jpg]

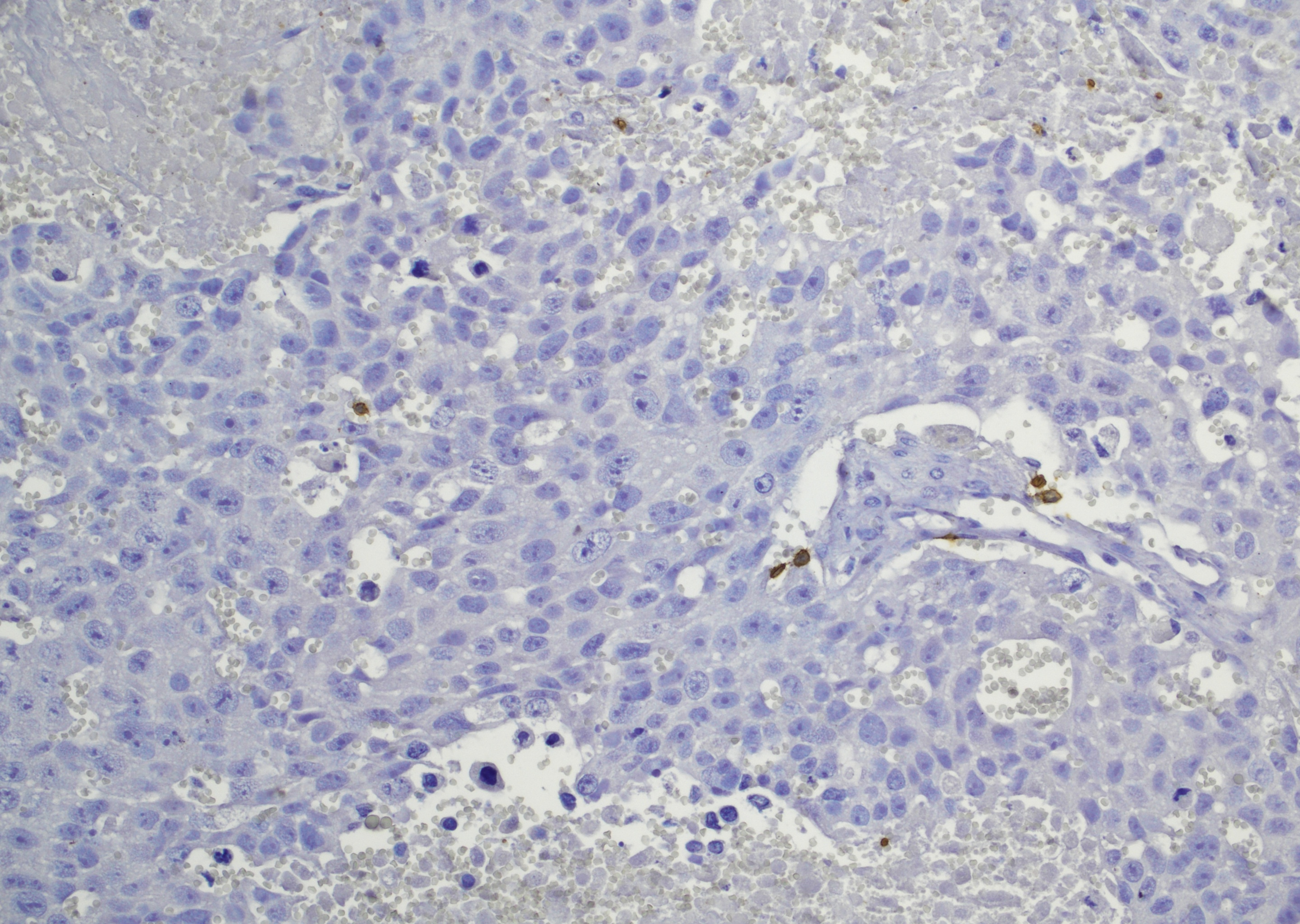

Supplement: Supplementary file 7 — Source Data Fig. 4 [file 44318_2024_40_MOESM7_ESM.zip › Figure 4/4H/4H B220 MM 20x.jpg]
