# Supplementary material for: Sterile inflammation via TRPM8 RNA-dependent TLR3-NF-kB/IRF3 activation promotes antitumor immunity in prostate cancer
Source: EMBO J. 2024 Feb 5;43(5):6. doi: 10.1038/s44318-024-00040-5 (PMC10907604; doi:10.1038/s44318-024-00040-5)

Fig EV1A

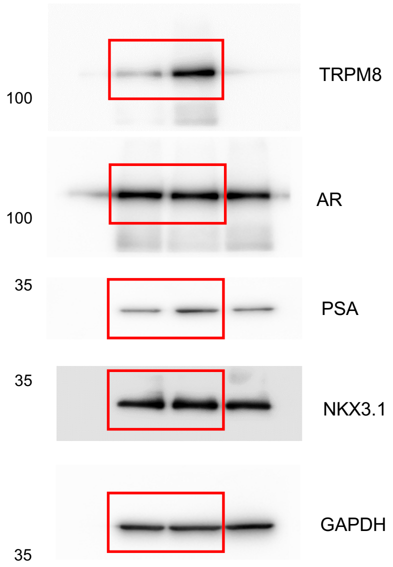

Fig EV1D

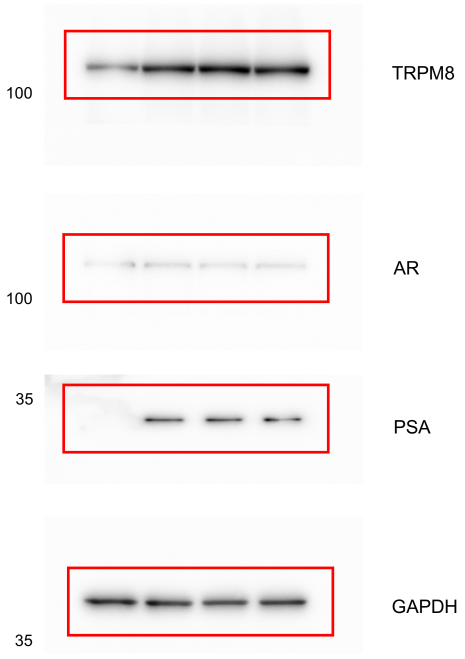

Fig EV2

Fig EV2A

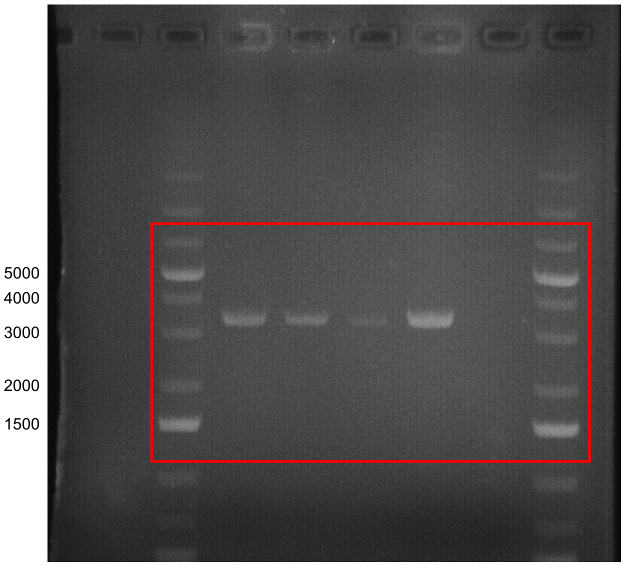

Fig EV2B

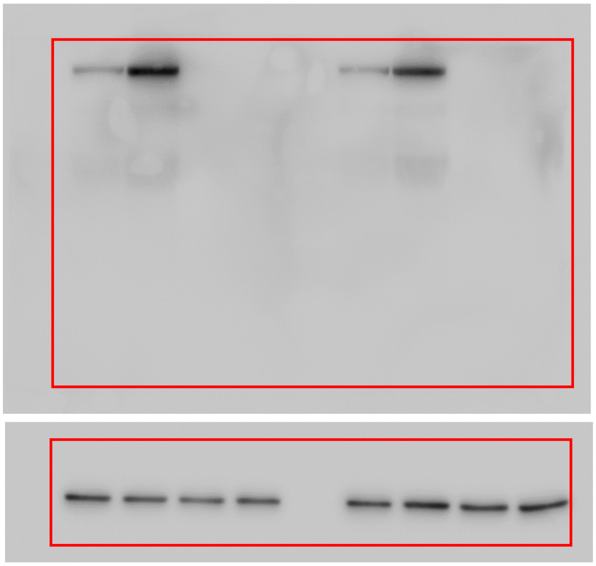

Fig EV2C

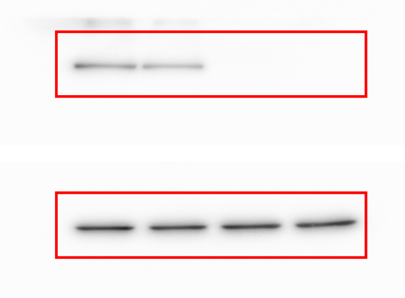

Fig EV2F

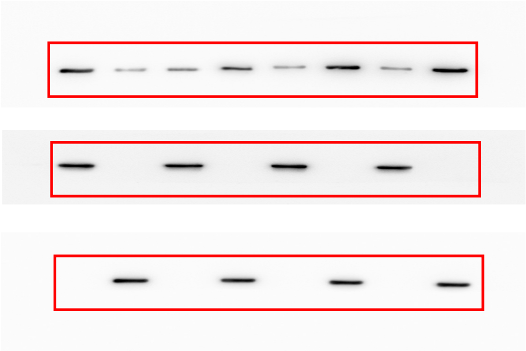

Fig EV3

Fig EV3A

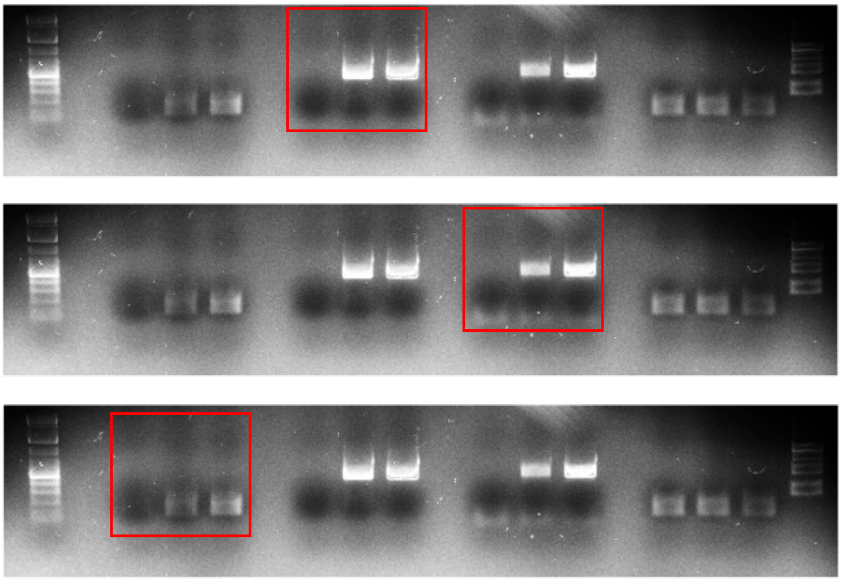

Fig EV3B

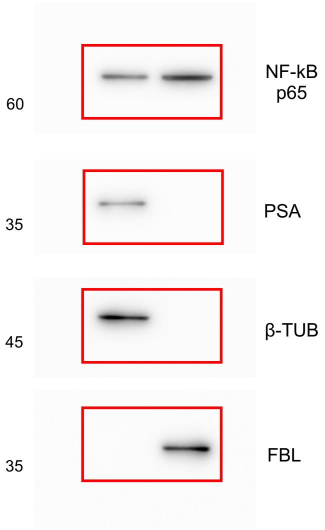

Fig EV3C

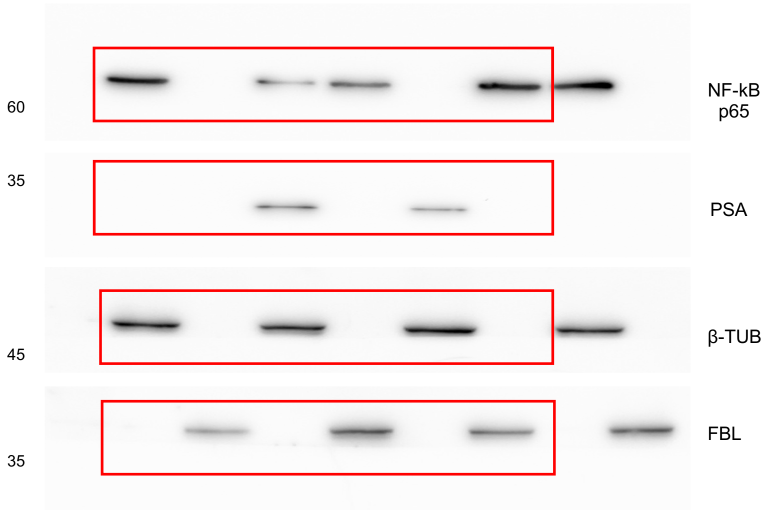

Fig EV3D

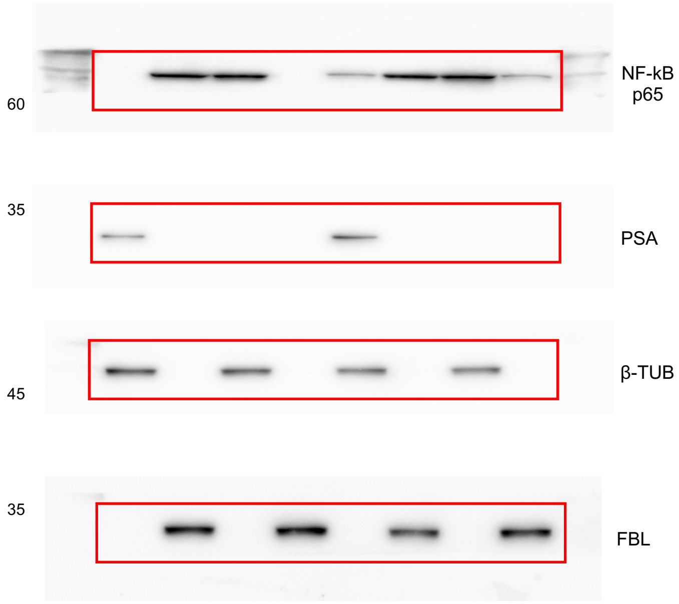

Fig EV3E

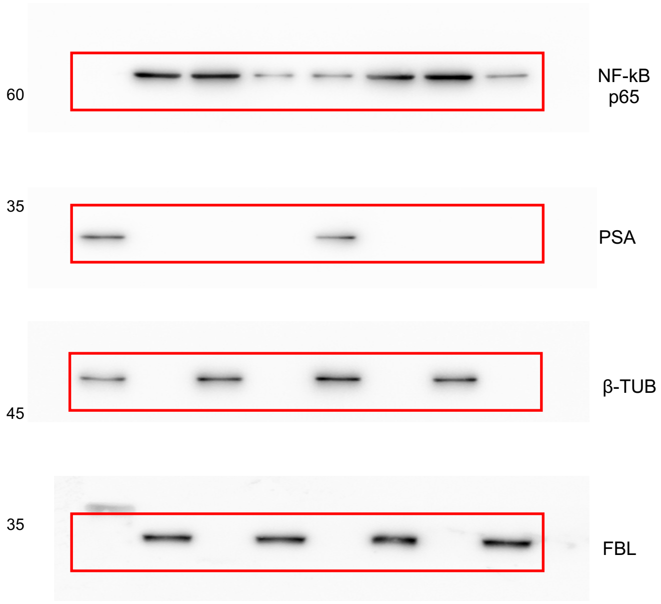

Fig EV4

Fig EV4D

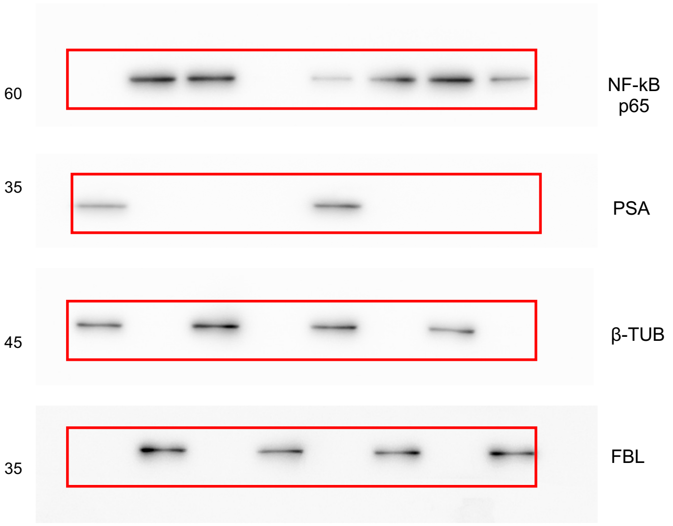

Fig EV4E

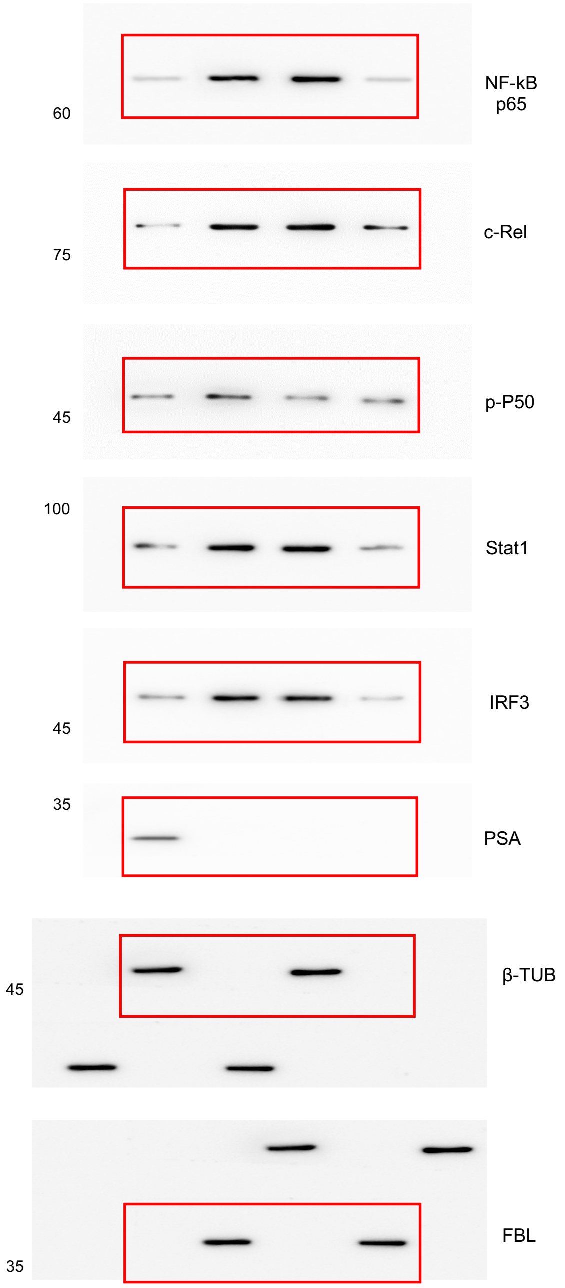

Fig EV4F

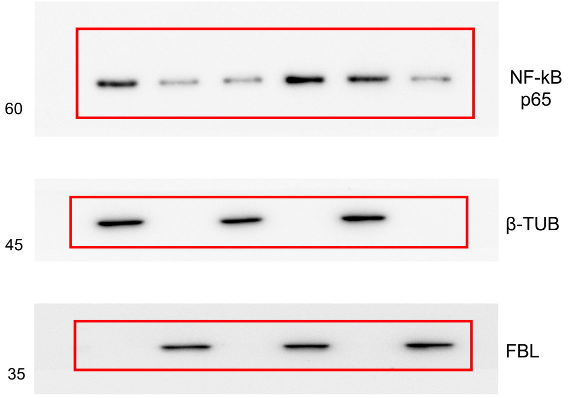

Fig EV5

Fig EV5F

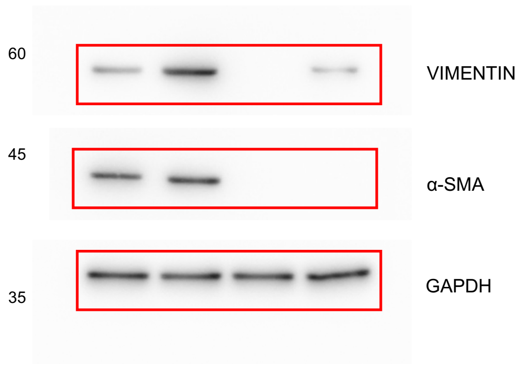

Fig EV5J

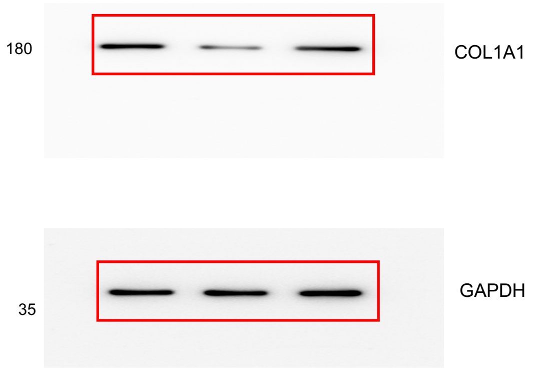

Supplement: Supplementary file 8 — Figure EV1-5 Source Data [file 44318_2024_40_MOESM8_ESM.zip › Figure EV 1-5/EV Uncropped Gels RR2Jan2024.pdf]
