# Supplementary material for: Sterile inflammation via TRPM8 RNA-dependent TLR3-NF-kB/IRF3 activation promotes antitumor immunity in prostate cancer
Source: EMBO J. 2024 Feb 5;43(5):6. doi: 10.1038/s44318-024-00040-5 (PMC10907604; doi:10.1038/s44318-024-00040-5)
Supplement: Supplementary file 10 — Expanded View Figures [file 44318_2024_40_MOESM10_ESM.pdf]

Expanded View Figures

Figure EV1. TRPM8 favors KLK3 expression in prostate cells.

(**A**) Immunoblot analysis showing expression of TRPM8, PSA and the other indicated proteins in LNCaP wild type (WT) and stably overexpressing TRPM8 (M8). GAPDH was used as loading control. (**B**) List of genes ranked by correlation coefficient whose expression in cancer correlates with *TRPM8* expression (TCGA, cBioportal). (**C**,**D**) RT-qPCR (**C**) and Western blot (**D**) analyses of *TRPM8* and *KLK3* (PSA) expression in RWPE-1 wild type (WT) and in three independent clones of RWPE-1 stably overexpressing TRPM8 (M8.1; M8.2; M8.3). GAPDH was analyzed after stripping of the primary and secondary antibodies used for PSA. (**E**) RT-qPCR analysis of RWPE-1 and RWPE-1 M8 cells treated with 100 nM Enzalutamide for 48 h. The Ct values associated with *KLK3* expression in RWPE-1 (34.33, 34.56, 34.72) confirmed the negligible expression of *KLK3* gene in these cells. Data Information: In (**C**,**E**) data are presented as mean ± SD of $n = 3$ independent biological replicates. *$P \leq 0.05$; **$P \leq 0.01$; ***$P \leq 0.001$; ns, not statistically significant (Two-tailed Student's *t*-test). Source data are available online for this figure.

© The Author(s)

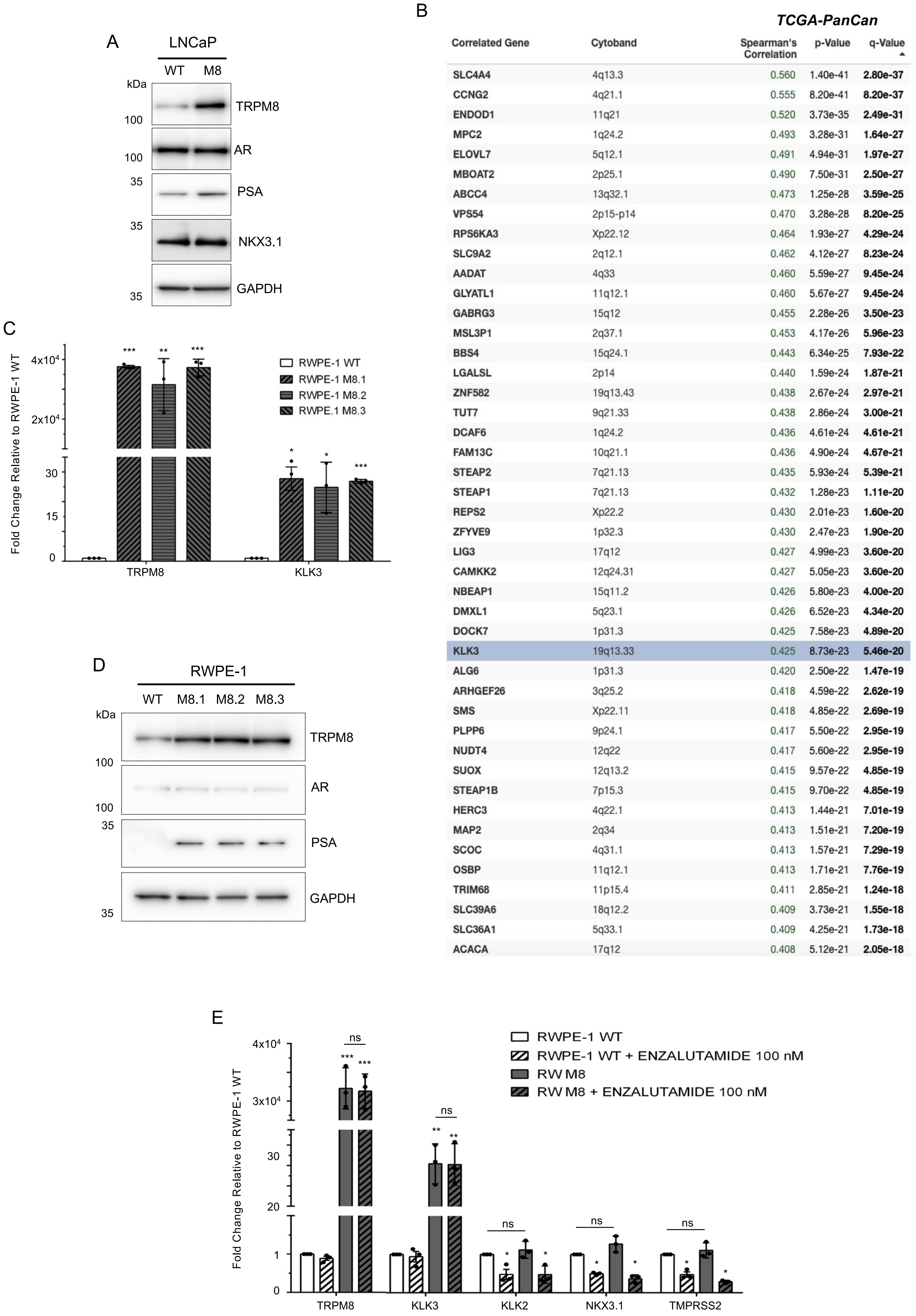

TCGA-PanCan

Correlated Gene	Cytoband	Spearman's Correlation	p-Value	q-Value
SLC4A4	4q13.3	0.560	1.40e-41	2.80e-37
CCNG2	4q21.1	0.555	8.20e-41	8.20e-37
ENDOD1	11q21	0.520	3.73e-35	2.49e-31
MPC2	1q24.2	0.493	3.28e-31	1.64e-27
ELOVL7	5q12.1	0.491	4.94e-31	1.97e-27
MBOAT2	2p25.1	0.490	7.50e-31	2.50e-27
ABCC4	13q32.1	0.473	1.25e-28	3.59e-25
VPS54	2p15-p14	0.470	3.28e-28	8.20e-25
RPS6KA3	Xp22.12	0.464	1.93e-27	4.29e-24
SLC9A2	2q12.1	0.462	4.12e-27	8.23e-24
AADAT	4q33	0.460	5.59e-27	9.45e-24
GLYATL1	11q12.1	0.460	5.67e-27	9.45e-24
GABRG3	15q12	0.455	2.28e-26	3.50e-23
MSL3P1	2q37.1	0.453	4.17e-26	5.96e-23
BBS4	15q24.1	0.443	6.34e-25	7.93e-22
LGALSL	2p14	0.440	1.59e-24	1.87e-21
ZNF582	19q13.43	0.438	2.67e-24	2.97e-21
TUT7	9q21.33	0.438	2.86e-24	3.00e-21
DCAF6	1q24.2	0.436	4.61e-24	4.61e-21
FAM13C	10q21.1	0.436	4.90e-24	4.67e-21
STEAP2	7q21.13	0.435	5.93e-24	5.39e-21
STEAP1	7q21.13	0.432	1.28e-23	1.11e-20
REPS2	Xp22.2	0.430	2.01e-23	1.60e-20
ZFYVE9	1p32.3	0.430	2.47e-23	1.90e-20
LIG3	17q12	0.427	4.99e-23	3.60e-20
CAMKK2	12q24.31	0.427	5.05e-23	3.60e-20
NBEAP1	15q11.2	0.426	5.80e-23	4.00e-20
DMXL1	5q23.1	0.426	6.52e-23	4.34e-20
DOCK7	1p31.3	0.425	7.58e-23	4.89e-20
KLK3	19q13.33	0.425	8.73e-23	5.46e-20
ALG6	1p31.3	0.420	2.50e-22	1.47e-19
ARHGEF26	3q25.2	0.418	4.59e-22	2.62e-19
SMS	Xp22.11	0.418	4.85e-22	2.69e-19
PLPP6	9p24.1	0.417	5.50e-22	2.95e-19
NUDT4	12q22	0.417	5.60e-22	2.95e-19
SUOX	12q13.2	0.415	9.57e-22	4.85e-19
STEAP1B	7p15.3	0.415	9.70e-22	4.85e-19
HERC3	4q22.1	0.413	1.44e-21	7.01e-19
MAP2	2q34	0.413	1.51e-21	7.20e-19
SCOC	4q31.1	0.413	1.57e-21	7.29e-19
OSBP	11q12.1	0.413	1.71e-21	7.76e-19
TRIM68	11p15.4	0.411	2.85e-21	1.24e-18
SLC39A6	18q12.2	0.409	3.73e-21	1.55e-18
SLC36A1	5q33.1	0.409	4.25e-21	1.73e-18
ACACA	17q12	0.408	5.12e-21	2.05e-18

 © The Author(s)

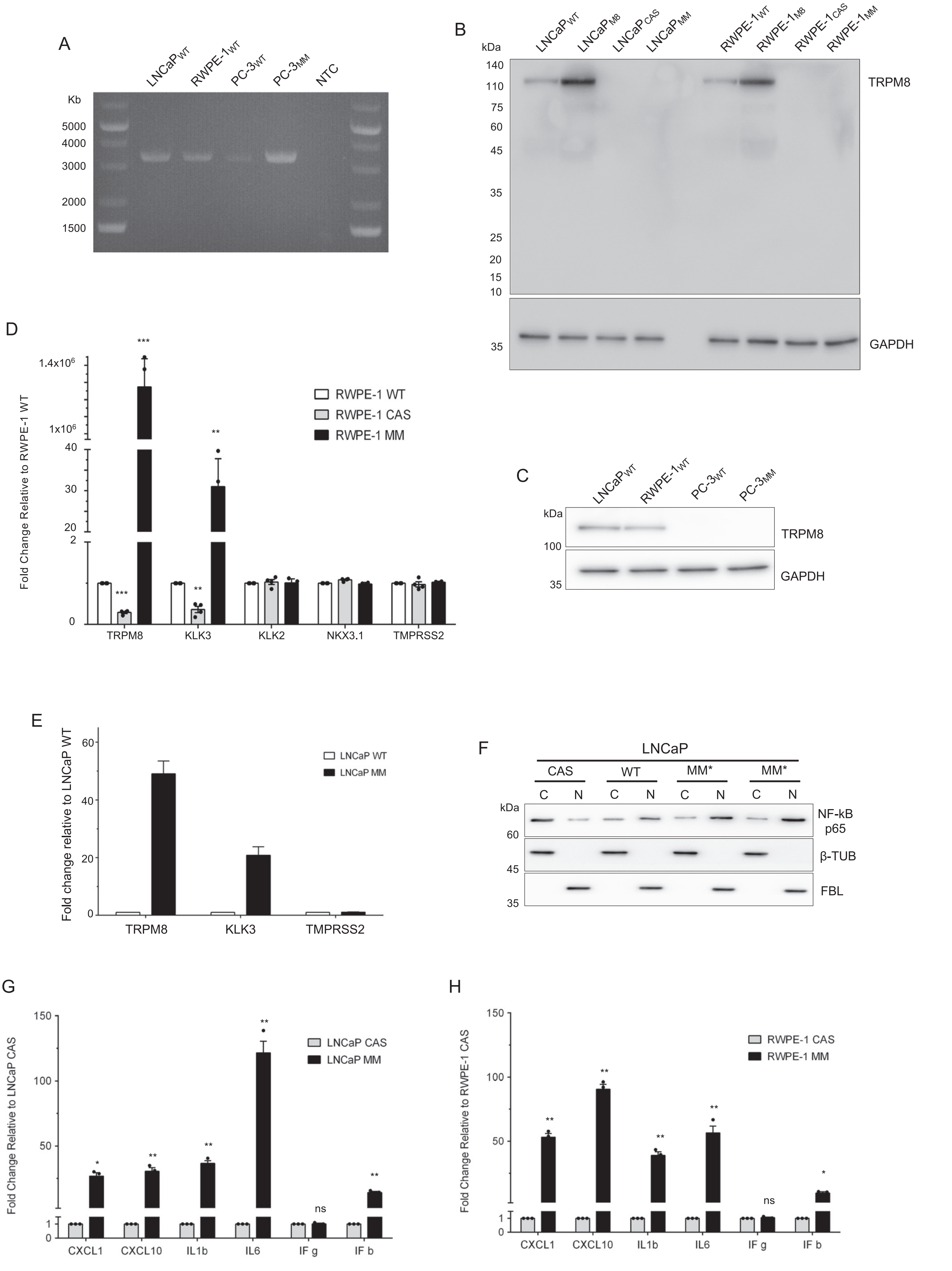
A
LNCaPWT
RWPE-1WT
PC-3WT
PC-3MM
NTC
Kb
5000
4000
3000
2000
1500
B
kDa
LNCaPWT
LNCaPM8
LNCaPCAS
LNCaPMM
RWPE-1WT
RWPE-1M8
RWPE-1CAS
RWPE-1MM
140
110
75
60
45
35
25
20
15
10
TRPM8
35
GAPDH
C
LNCaPWT
RWPE-1WT
PC-3WT
PC-3MM
kDa
100
TRPM8
35
GAPDH
D
Fold Change Relative to RWPE-1 WT
1.4x10^6
1x10^6
40
30
20
2
0
RWPE-1 WT
RWPE-1 CAS
RWPE-1 MM
***
**
TRPM8
KLK3
KLK2
NKX3.1
TMPRSS2
E
Fold change relative to LNCaP WT
60
40
20
0
LNCaP WT
LNCaP MM
TRPM8
KLK3
TMPRSS2
F
LNCaP
CAS
WT
MM*
MM*
C
N
kDa
60
45
35
NF-kB p65
β-TUB
FBL
G
Fold Change Relative to LNCaP CAS
150
100
50
1
0
LNCaP CAS
LNCaP MM
*
**
ns
CXCL1
CXCL10
IL1b
IL6
IF g
IF b
H
Fold Change Relative to RWPE-1 CAS
150
100
50
1
0
RWPE-1 CAS
RWPE-1 MM
**
*
ns
CXCL1
CXCL10
IL1b
IL6
IF g
IF b

© The Author(s)

Figure EV2. Pro-inflammatory activity of the mutant non-coding RNA of TRPM8.

(**A**,**B**) End-point PCR (**A**) and Western blot analysis (**B**) of TRPM8 in LNCaP (WT), RWPE-1 (WT) and PC-3 (WT and MM) cell lines. GAPDH was used as loading control. (**C**) Immunoblotting analysis of TRPM8 protein with the Alomone (#ACC-049) antibody in wild type and genetically modified (M8, CAS, MM) LNCaP and RWPE-1 cell lines. (**D**) RT-qPCR analysis of *TRPM8*, *KLK3* and *TMPRSS2* genes in both wild type (WT) and genetically engineered (MM, two independent clones) LNCaP cell lines expressing reduced levels of TRPM8 RNA compared to clones described in Fig. 1C. (**E**) Immunoblot analysis of NF-kB p65 in cytosolic and nuclear fractions of CAS, WT, and MM (described in **D**) engineered LNCaP cell lines. β-Tubulin (β-TUB) and Fibrillarin (FBL) were used as cytoplasmic and nuclear markers, respectively. (**F**,**G**) RT-qPCR analyses of canonical NF-kB and IRF3 targets genes in both LNCaP and RWPE-1 CAS and MM cells. Data Information: In (**D**,**E**,**G**,**H**) data are presented as mean ± SD of $n = 4$ (**D**), $n = 2$ (**E**), and $n = 3$ (**G**,**H**) independent biological replicates. *$P \leq 0.05$; **$P \leq 0.01$; ns, not statistically significant (Two-tailed Student's *t*-test). Source data are available online for this figure.

 © The Author(s)

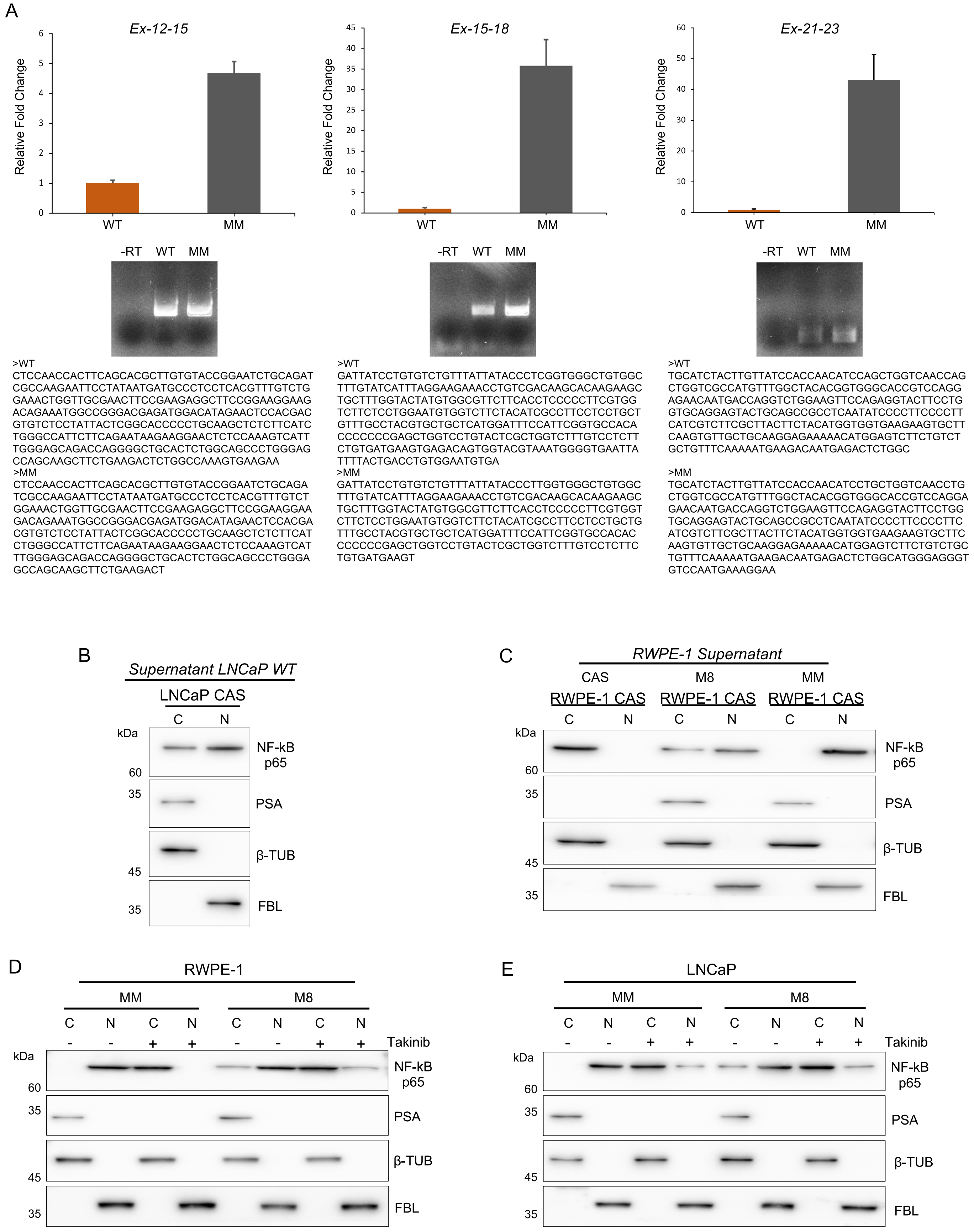
A
Ex-12-15
Ex-15-18
Ex-21-23
Relative Fold Change
WT
MM
-RT WT MM
>WT
CTCCAACCACTTCAGCACGCTTGTGTACCGGAATCTGCAGAT
CGCCAAGAATTCCTATAATGATGCCCTCCTCACGTTTGTCTG
GAAACTGGTTGCGAACTTCCGAAGAGGCTTCCGGAAGGAAG
ACAGAAATGGCCGGGACGAGATGGACATAGAACTCCACGAC
GTGTCTCCTATTACTCGGCACCCCCTGCAAGCTCTCTTCATC
TGGGCCATTCTTCAGAATAAGAAGGAACTCTCCAAAGTCATT
TGGGAGCAGACCAGGGGCTGCACTCTGGCAGCCCTGGGAG
CCAGCAAGCTTCTGAAGACTCTGGCCAAAGTGAAGAA
>MM
CTCCAACCACTTCAGCACGCTTGTGTACCGGAATCTGCAGA
TCGCCAAGAATTCCTATAATGATGCCCTCCTCACGTTTGTCT
GGAAACTGGTTGCGAACTTCCGAAGAGGCTTCCGGAAGGAA
GACAGAAATGGCCGGGACGAGATGGACATAGAACTCCACGA
CGTGTCTCCTATTACTCGGCACCCCCTGCAAGCTCTCTTCAT
CTGGGCCATTCTTCAGAATAAGAAGGAACTCTCCAAAGTCAT
TTGGGAGCAGACCAGGGGCTGCACTCTGGCAGCCCTGGGA
GCCAGCAAGCTTCTGAAGACT
>WT
GATTATCCTGTGTCTGTTTATTATACCCTCGGTGGGCTGTGGC
TTTGTATCATTTAGGAAGAAACCTGTCGACAAGCACAAGAAGC
TGCTTTGGTACTATGTGGCGTTCTTCACCTCCCCCTTCGTGG
TCTTCTCCTGGAATGTGGTCTTCTACATCGCCTTCCTCCTGCT
GTTTGCCTACGTGCTGCTCATGGATTTCCATTCGGTGCCACA
CCCCCCCGAGCTGGTCCTGTACTCGCTGGTCTTTGTCCTCTT
CTGTGATGAAGTGAGACAGTGGTACGTAAATGGGGTGAATTA
TTTTACTGACCTGTGGAATGTGA
>MM
GATTATCCTGTGTCTGTTTATTATACCCTTGGTGGGCTGTGGC
TTTGTATCATTTAGGAAGAAACCTGTCGACAAGCACAAGAAGC
TGCTTTGGTACTATGTGGCGTTCTTCACCTCCCCCTTCGTGGT
CTTCTCCTGGAATGTGGTCTTCTACATCGCCTTCCTCCTGCTG
TTTGCCTACGTGCTGCTCATGGATTTCCATTCGGTGCCACAC
CCCCCCGAGCTGGTCCTGTACTCGCTGGTCTTTGTCCTCTTC
TGTGATGAAGT
>WT
TGCATCTACTTGTTATCCACCAACATCCAGCTGGTCAACCAG
CTGGTCGCCATGTTTGGCTACACGGTGGGCACCGTCCAGG
AGAACAATGACCAGGTCTGGAAGTTCCAGAGGTACTTCCTG
GTGCAGGAGTACTGCAGCCGCCTCAATATCCCCTTCCCCTT
CATCGTCTTCGCTTACTTCTACATGGTGGTGAAGAAGTGCTT
CAAGTGTTGCTGCAAGGAGAAAAACATGGAGTCTTCTGTCT
GCTGTTTCAAAAATGAAGACAATGAGACTCTGGC
>MM
TGCATCTACTTGTTATCCACCAACATCCTGCTGGTCAACCTG
CTGGTCGCCATGTTTGGCTACACGGTGGGCACCGTCCAGGA
GAACAATGACCAGGTCTGGAAGTTCCAGAGGTACTTCCTGG
TGCAGGAGTACTGCAGCCGCCTCAATATCCCCTTCCCCTTC
ATCGTCTTCGCTTACTTCTACATGGTGGTGAAGAAGTGCTTC
AAGTGTTGCTGCAAGGAGAAAAACATGGAGTCTTCTGTCTGC
TGTTTCAAAAATGAAGACAATGAGACTCTGGCATGGGAGGGT
GTCCAATGAAAGGAA
B
Supernatant LNCaP WT
LNCaP CAS
C N
kDa
NF-kB p65
PSA
β-TUB
FBL
C
RWPE-1 Supernatant
CAS M8 MM
RWPE-1 CAS RWPE-1 CAS RWPE-1 CAS
C N C N C N
NF-kB p65
PSA
β-TUB
FBL
D
RWPE-1
MM M8
C N C N C N C N
Takinib
NF-kB p65
PSA
β-TUB
FBL
E
LNCaP
MM M8
C N C N C N C N
Takinib
NF-kB p65
PSA
β-TUB
FBL

Figure EV3. TRPM8 RNA secretion modulates TLR3/TAK1 signal cascade.

(**A**) RT-qPCR analysis of TRPM8 transcript released by WT and MM LNCaP cells into extracellular vesicles (EVs). Specificity and sequence of amplicons obtained with sets of primers spanning different exons (Ex) of the coding sequence of TRPM8 RNA are shown. *Y3* transcript was used to normalize the data. (**B**) Nucleus/Cytosol fractionation and Western blot analysis of NF-kB p65 and PSA in LNCaP CAS cells conditioned with the supernatants of LNCaP WT cells. β-Tubulin (β-TUB) and Fibrillarin (FBL) were used as markers of the cytosolic and nuclear fractions, respectively. (**C**) Nucleus/Cytosol fractionation and Western blot analysis of NF-kB p65 and PSA in RWPE-1 CAS cells conditioned with the supernatants of RWPE-1 (CAS, M8, and MM). β-Tubulin (β-TUB) and Fibrillarin (FBL) were used as markers of the cytosolic and nuclear fractions, respectively. (**D**,**E**) Nucleus/Cytosol fractionation and Western blot analysis of NF-kB p65 and PSA in RWPE-1 MM and M8 (**D**) and LNCaP MM and M8 (**E**) cell lines in the presence or absence of Takinib (TAK1 inhibitor; 10 μM, 24 h). β-Tubulin (β-TUB) and Fibrillarin (FBL) were used as markers of the cytosolic and nuclear fractions, respectively. Data Information: In (**A**) data are presented as mean ± SD of $n = 3$ biological replicates. Source data are available online for this figure.

© The Author(s)

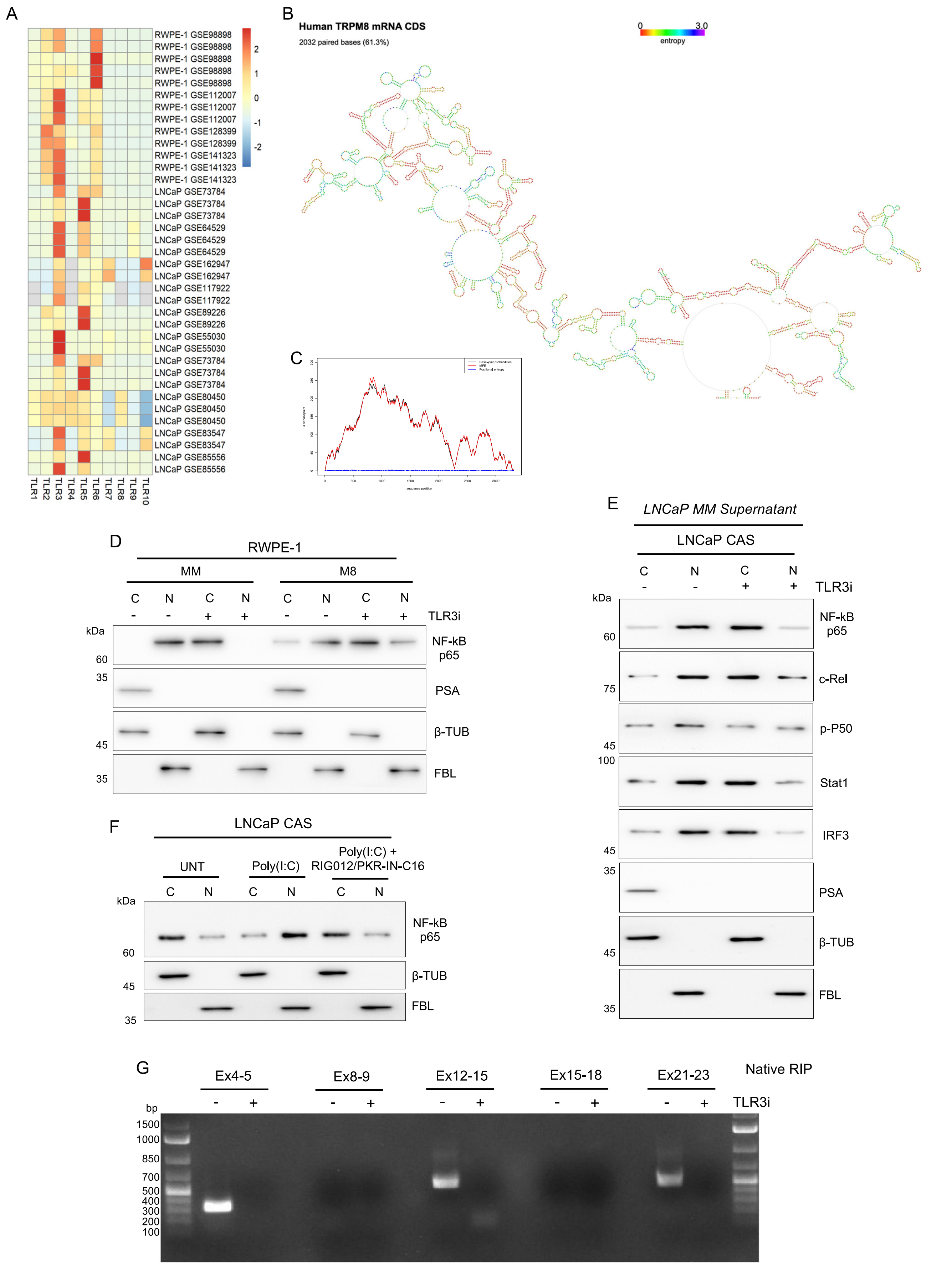
A
B
Human TRPM8 mRNA CDS
2032 paired bases (61.3%)
entropy
C
D
RWPE-1
MM
M8
TLR3i
NF-kB p65
PSA
β-TUB
FBL
E
LNCaP MM Supernatant
LNCaP CAS
TLR3i
NF-kB p65
c-Rel
p-P50
Stat1
IRF3
PSA
β-TUB
FBL
F
LNCaP CAS
UNT
Poly(I:C)
Poly(I:C) + RIG012/PKR-IN-C16
NF-kB p65
β-TUB
FBL
G
Ex4-5
Ex8-9
Ex12-15
Ex15-18
Ex21-23
Native RIP
TLR3i

Figure EV4. Prediction of TRPM8 RNA structures highlights putative TLR3 binding motifs.

(**A**) TLRs expression in LNCaP and RWPE-1 prostate cell lines based on publicly available datasets. (**B**) In silico prediction of the secondary structure of human TRPM8 transcript. Highly stable dsRNA stems with bulge/internal loops are indicated in red. (**C**) Mountain plot of (**B**). (**D**) Nucleus/Cytosol fractionation and Western blot analysis of NF-kB p65 and PSA in RWPE-1 MM and M8 cell lines in the presence or absence of TLR3/dsRNA Complex Inhibitor (TLR3 inhibitor; 20 μM, 24 h). β-Tubulin (β-TUB) and Fibrillarin (FBL) were used as markers of the cytosolic and nuclear fractions, respectively. (**E**) Nucleus/Cytosol fractionation and Western blot analysis of NF-kB p65, c-Rel, phospho-p50, STAT1, IRF3 and PSA in LNCaP CAS cells conditioned with the supernatants of LNCaP MM cells. β-Tubulin (β-TUB) and Fibrillarin (FBL) were used as markers of the cytosolic and nuclear fractions, respectively. (**F**) Nucleus/Cytosol fractionation and Western blot analysis of NF-kB p65 in LNCaP CAS cells transfected with Poly(I:C) (2 μg/mL, 24 h) and treated or not with the the combination of the RIG1 inhibitor RIG012 (2 μM, 6 h) and the PKR inhibitor C16 (2 μM, 6 h). Untransfected and untreated LNCaP CAS cells served as control for the activation of NF-kB by Poly(I:C). β-Tubulin (β-TUB) and Fibrillarin (FBL) were used as markers of the cytosolic and nuclear fractions, respectively. (**G**) PCR analysis of TLR3-bound TRPM8 transcript (native RIP) in LNCaP WT cells treated or not with TLR3/dsRNA complex inhibitor. Sets of primers spanning different exons (Ex) of the coding sequence of TRPM8 RNA are shown. Data Information: In (**A**) the heatmap was plotted with the pheatmap package, scaling gene expression by row. All the analyses were done with R 4.0.3. Source data are available online for this figure.

 © The Author(s)

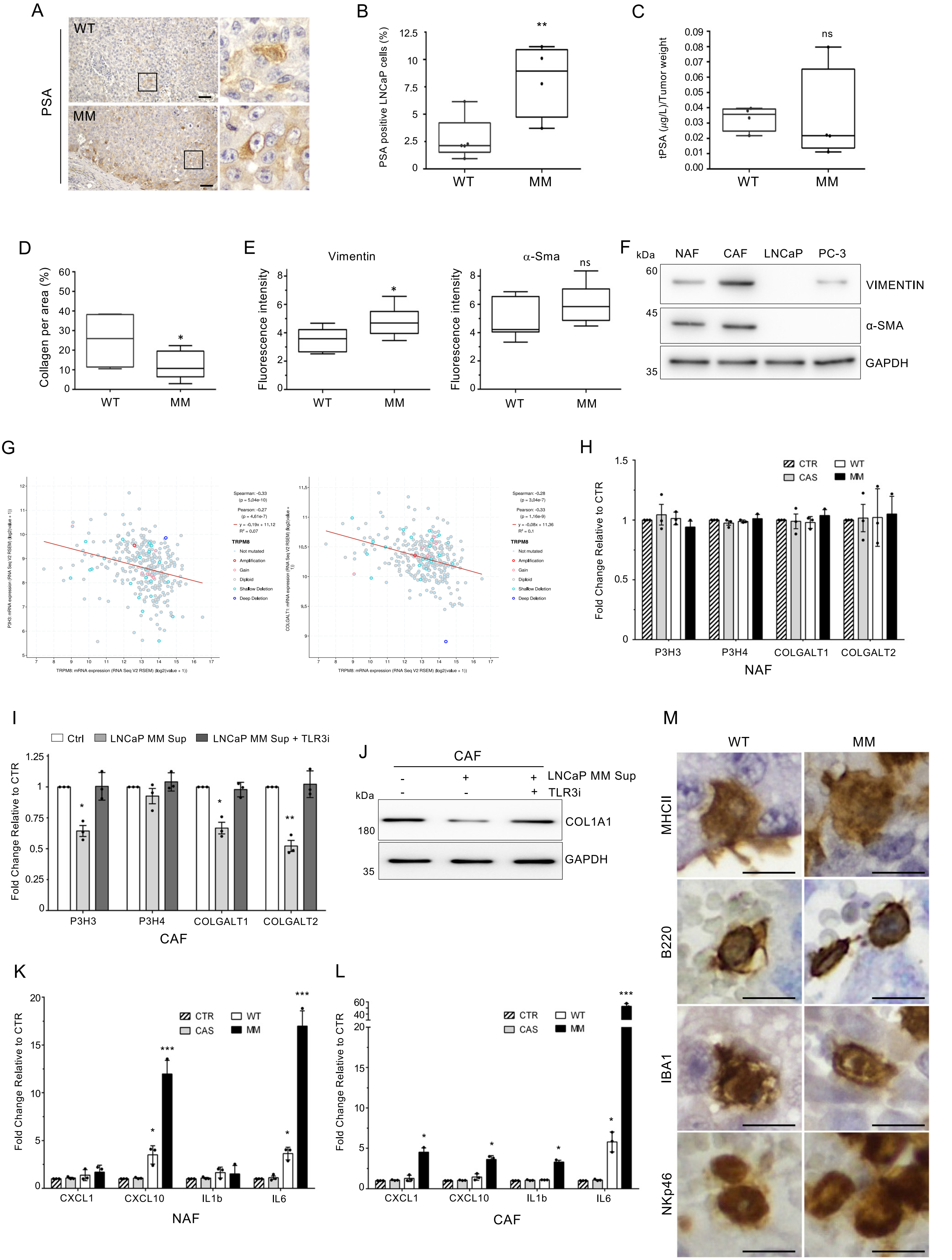
A
B
C
D
E
F
G
H
I
J
K
L
M
PSA
WT
MM
PSA positive LNCaP cells (%)
tPSA (μg/L)/Tumor weight
Collagen per area (%)
Vimentin
α-Sma
Fluorescence intensity
kDa
NAF
CAF
LNCaP
PC-3
VIMENTIN
α-SMA
GAPDH
Fold Change Relative to CTR
CTR
CAS
P3H3
P3H4
COLGALT1
COLGALT2
Ctrl
LNCaP MM Sup
LNCaP MM Sup + TLR3i
COL1A1
TLR3i
CXCL1
CXCL10
IL1b
IL6
MHCII
B220
IBA1
NKp46

Figure EV5. TRPM8 RNA secretion alters fibroblasts functions.

(**A**,**B**) Immunolocalization of PSA in FFPE sections of LNCaP WT (*n* = 3 xenografts/5 sections) and MM (*n* = 2 xenografts/4 sections) tumors (**A**), coupled with the percentage of PSA+ cells (total number of cells counted: WT = 142.293; MM = 106.013) (**B**). (**C**) Quantification of total PSA (free and complexed) circulating in the plasma of xenografted mice (WT, *n* = 4; MM, *n* = 4). (**D**) Quantification of Collagen Type-I (percentage of blue area to total section area; *n* = 6 sections per genotype), relative to Fig. 4D. (**E**) Quantification of Vimentin and α-Sma in FFPE sections of LNCaP WT and LNCaP MM xenograft (a minimum of 5 different areas per genotype were analyzed), relative to Fig. 4E. (**F**) Western blot analysis of Vimentin and α-SMA proteins in NAF, CAF, LNCaP and PC-3 cell lines. GAPDH was used as loading control. (**G**) Inverse correlation in PCa of the expression of *P3H3* (left panel) and *COLGALT1* (right panel) with respect to *TRPM8* (TCGA, cBioportal). (**H**) RT-qPCR analysis of the indicated genes of the metabolism of type I collagen in NAF cells. (**I**) RT-qPCR analysis of *PH3*, *PH4*, *COLGALT1*, and *COLGALT2* genes expression in CAFs conditioned with the supernatant of LNCaP MM cells and treated or not with the TLR3 inhibitor (20 μM, 24 h). (**J**) Western blot analysis of COL1A1 levels in CAFs conditioned with the supernatant of LNCaP MM cells and treated or not with the TLR3 inhibitor (XX μM, 24 h). GAPDH was used as loading control. (**K**,**L**) RT-qPCR analysis of canonical NF-kB targets genes in NAF (**K**) and CAF (**L**) cells conditioned with the supernatants of LNCaP (CAS, WT, MM) cell lines. (**M**) Magnification images of MHCII, CD45R (B220), IBA1 and NKp46 immunolocalization in FFPE sections of LNCaP wild type (WT) and LNCaP MM xenografts. Scale bars, 20 μm. Data Information: In (**B**–**E**) box-plots elements indicate the median (center line), upper and lower quartiles (box limits). Whiskers extend from the minimum to the maximum. *$P \le 0.05$; **$P \le 0.01$. (Student's *t*-test). In (**H**,**I**,**K**,**L**) data are presented as mean ± SD of *n* = 3 independent biological replicates. *$P \le 0.05$; **$P \le 0.01$; ***$P \le 0.001$. (Two-tailed Student's *t*-test). Source data are available online for this figure.

 © The Author(s)
